# Targeting strategies for bone diseases: signaling pathways and clinical studies

**DOI:** 10.1038/s41392-023-01467-8

**Published:** 2023-05-17

**Authors:** Hao Xu, Wentao Wang, Xin Liu, Wei Huang, Chen Zhu, Yaozeng Xu, Huilin Yang, Jiaxiang Bai, Dechun Geng

**Affiliations:** 1 Department of Orthopedics, The First Affiliated Hospital of Soochow University, 188 Shizi Street, Suzhou, Jiangsu 215006 P. R. China; 2 Department of Orthopedics, The First Affiliated Hospital of USTC, Division of Life Sciences and Medicine, University of Science and Technology of China, Hefei, 230031 Anhui China; 3 Orthopaedic Institute, Medical College, Soochow University, Suzhou, 215006 Jiangsu China

**Keywords:** Drug delivery, Translational research

## Abstract

Since the proposal of Paul Ehrlich’s magic bullet concept over 100 years ago, tremendous advances have occurred in targeted therapy. From the initial selective antibody, antitoxin to targeted drug delivery that emerged in the past decades, more precise therapeutic efficacy is realized in specific pathological sites of clinical diseases. As a highly pyknotic mineralized tissue with lessened blood flow, bone is characterized by a complex remodeling and homeostatic regulation mechanism, which makes drug therapy for skeletal diseases more challenging than other tissues. Bone-targeted therapy has been considered a promising therapeutic approach for handling such drawbacks. With the deepening understanding of bone biology, improvements in some established bone-targeted drugs and novel therapeutic targets for drugs and deliveries have emerged on the horizon. In this review, we provide a panoramic summary of recent advances in therapeutic strategies based on bone targeting. We highlight targeting strategies based on bone structure and remodeling biology. For bone-targeted therapeutic agents, in addition to improvements of the classic denosumab, romosozumab, and PTH1R ligands, potential regulation of the remodeling process targeting other key membrane expressions, cellular crosstalk, and gene expression, of all bone cells has been exploited. For bone-targeted drug delivery, different delivery strategies targeting bone matrix, bone marrow, and specific bone cells are summarized with a comparison between different targeting ligands. Ultimately, this review will summarize recent advances in the clinical translation of bone-targeted therapies and provide a perspective on the challenges for the application of bone-targeted therapy in the clinic and future trends in this area.

## Introduction

Bone is a solid structure undergoing perpetual renewal with crucial functions such as kinematic support, visceral protection, and regulation of hematopoiesis and mineral balance.^1^ Maintenance of these functions depends on normal bone mass and strength, which are achieved through bone remodeling. Skeletal diseases, such as osteoporosis, are usually accompanied by abnormal bone remodeling, in which osteoclast-mediated bone resorption preponderates over osteoblast-mediated bone formation, leading to decreased bone mass, deteriorated microstructure, and increased fragile fracture risk.^2^ In light of the perplexing pathological mechanism underlying abnormal remodeling, symptom-relief therapy by anti-resorption and pro-formation is the main choice, in addition to the elemental calcium and vitamin D supplements.

Since the proposal of Paul Ehrlich’s “magic bullet” concept over 100 years ago, great advances have occurred in drugs that target intended cellular structures.^3^ In bone-targeted pharmacological therapy, anti-resorption agents, such as bisphosphonates, selective estrogen receptor modulators (SERMs), receptor activator of nuclear factor-kB (RANK) ligand (RANKL) inhibitors, and anabolic medications, such as type 1 parathyroid hormone receptor (PTH1R) ligands, sclerostin inhibitors, have emerged with demonstrated efficacy in treating diseases characterized by abnormal bone remodeling.^4,5^ Nevertheless, the improvement in bone parameters by these agents does not simply imply a regain of normal bone remodeling, as is observed by the attendant suppression of anti-resorption agents to the bone formation or a slight increase in bone resorption by anabolic agents.^6,7^ Furthermore, side effects such as osteonecrosis,^8^ rebound fractures,^9^ cardiovascular events,^10^ and osteosarcoma genesis,^11^ impede effective long-term management of bone diseases, which underscores a need to improve these established targets for more precise therapy.

Fortunately, with the deepening knowledge of bone biology, the mechanism underlying some side effects has been recognized with a prominent decrease by preclinical improvements of these drugs. In addition, recent insights have revealed that bone remodeling is a coordinated process spatiotemporally mediated by all bone cells instead of single activities by the basic multicellular units consisting of osteoblasts (OBs) and osteoclasts (OCs).^12^ Osteocytes, immune cells, vessel endothelial cells, and bone marrow cells were shown to possess multiple influences during the remodeling process,^13–15^ and some emerging therapeutic strategies targeting these bone cells, especially the crosstalk between them, have shown promise in promoting bone homeostasis in preclinical studies, which may facilitate the development of new drug targets.

Nevertheless, the term “targeted drugs” here refers specifically to drugs acting on the intended therapeutic sites. After administration, they are still distributed throughout the body and can affect other tissues and cells. In addition, the compactness and lessened blood flow of bone tissue further limit the osteotropism of drugs. Fortunately, since Pierce et al. first proposed the concept of “bone targeting” in 1986, a new era of ligand-based bone-targeted therapy was initiated.^16^ Multifarious drug delivery vectors ranging from the micron scale to the nanoscale, and superficial modifications, such as PEGylation, have emerged with decreased drug depletion from the reticuloendothelial system and increased circulation time. By further conjugating these ‘protected’ drugs with bone-targeted ligands, active osteotropism can be obtained with higher concentrations in bone, longer sustained and local release, and decreased minimal effective doses to realize authentic bone targeting.^17^ Thanks to the development of molecular biology techniques such as Cell-SELEX, bioorthogonal chemistry, and phage display, the selection of the targeting ligands is no longer confined to the initial hydroxyapatite-targeted ligands represented by bisphosphonates and tetracyclines.^18^ Aptamers, peptides, and other small molecule ligands with cell-specific affinity have driven bone targeting toward the cellular level.^19^ Additionally, a deeper understanding of some endogenous migration processes of specific cells has triggered some effective biomimetic delivery attempts.^20^ Although it remains challenging to determine the merits of bone tissue targeting versus bone cell targeting, both approaches have shown promising therapeutic effects in preclinical investigations.

In this review, we provide an all-round view of therapy strategies based on bone targeting. The targeting strategies for drugs and deliveries will be illustrated based on an introduction of bone remodeling biology advances. The clinical translations of them are also summarized and discussed. We expect this review to present useful information for a comprehensive understanding of bone-targeted therapy.

## Bone remodeling biology: signaling pathways and cellular crosstalk

### Osteoclasts

As individual bone-resorbing cells in the human body, osteoclasts differentiate from monocytes/macrophages of the hematopoietic lineage in the bone marrow. Under the guidance of sphingosine 1-phosphate (S1P) signaling, osteoclast precursors (pOCs) migrate to the bone resorption surface from bone marrow and circulation through the collagen network.^21^ Successful osteoclastogenesis (fusion of pOCs into multinucleated, mature osteoclasts (mOCs) with bone-resorbing ability) relies on RANKL and macrophage colony-stimulating factor (M-CSF) produced by osteogenic cells, T cells, and vascular endothelial cells near the bone surface.^22,23^ Soluble and membrane RANKL (sRANKL and mRANKL) binds to RANK on pOC membranes and triggers intracellular activation of tumor necrosis factor receptor-associated factor (TRAF) signaling, especially TRAF 2, 5, and 6, which further activates intracellular nuclear factor-kB (NF-κB) and mitogen-activated protein kinase (MAPK) signaling to induce myelocytomatosis viral oncogene homolog (MYC) and Fos proto-oncogene, AP-1 transcription factor subunit (FOS) expression, resulting in a nuclear factor of activated T-cell, c1 (NFATc1) signaling expression in the canonical signaling pathway.^24^

**Fig. 1:**
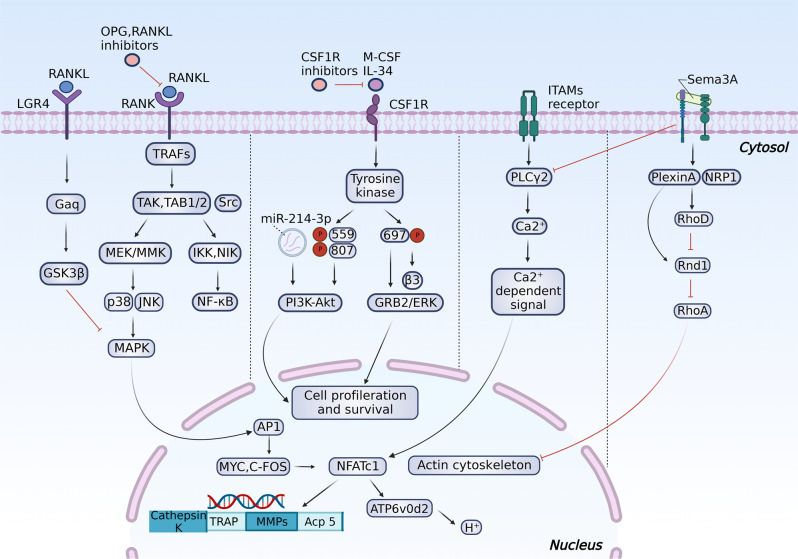
An overview of intracellular regulation of osteoclastogenesis and resorption activities. The early differentiation of myeloid progenitors to pOCs is mediated by M-CSF stimuli via PI3K/Akt and GRB2/ERK signaling. The binding of RANKL to RANK further promotes the differentiation of pOCs and activates intracellular TRAFs/NF-κB and TRAFs/MAPK signaling to increase transcription factors such as MYC, FOS, and NFATc1, upregulating the expression of osteoclast activation-related proteins and acid secretion. Phosphorylation of Plcγ2 by ITAM stimuli is also required for RANKL-induced NFATc1 activation.^468^ LGR4 activation can suppress RANKL-induced osteoclastogenesis via the GSK3β/MAPK pathway. Sema3A can inhibit ITAM-induced Plcγ2 activation and M-CSF-induced osteoclast differentiation through the RhoA signaling pathway

As a transcription factor, NFATc1 promotes osteoclastogenesis by upregulating the expression of resorption-related genes, such as cathepsin K, matrix metalloproteinase 9 (MMP9), tartrate-resistant acid phosphatase (TRAP), and acid phosphatase 5 (Acp5).^25,26^ Apart from RANK/RANKL and M-CSF, other factors have been revealed to participate in osteoclastogenesis. Wingless-type MMTV integration site family 5a (WNT5a) expressed by osteoblasts can stimulate the differentiation of pOCs in the noncanonical pathway by binding to the Frizzled (FZD)-receptor tyrosine kinase-like orphan receptor 2 (ROR2)^27^ and reverse the inhibitory effects of WNT16 on RANKL-induced osteoclastogenesis.^28^ Toll-like receptors and adapters containing immunoreceptor tyrosine-based activation motifs (ITAMs), such as Fc receptor common gamma subunit (FcRγ) and DNAX-activating protein (DAP) 12, are critical costimulatory receptors on pOCs that foster osteoclast maturation.^29–31^ In contrast, osteoclastogenesis inhibitory factor (OPG, also known as osteoprotegerin) and leucine-rich repeat containing G protein-coupled receptor 4 (LGR4, another RANKL receptor on osteoclast membrane) inhibit the process by binding RANK against RANKL and receiving RANKL against RANK, respectively (Fig. 1).^32,33^

mOCs are polarized, multinucleated cells that attach to the bone surface and generate a resorption lacuna by releasing lysosomal proteases, such as cathepsin K, TRAP, and MMPs.^34^ In the acidic lacuna with a pH of approximately 4.5,^35^ bone minerals and demineralized organic components are degraded, endocytosed, and released through the secretory domain at the opposite side of the cell.^36^ Successful attachment to the bone matrix and formation of the ruffled border is the initial phase of the resorption process, which depends on membrane integrin α5β1, α2β1, and α5β3-mediated polarization of osteoclasts.^37^ Among them, α5β3 is the most abundantly expressed and mediates the adhesion of osteoclasts to the bone matrix proteins such as osteopontin (OPN), bone sialoprotein, and fibronectin by recognizing Arg-Gly-Asp (RGD) motifs in the matrix.^38,39^ Then, the binding complex activates the adaptive change of the osteoclast’s apical membrane, and the formation of actin rings via the phospholipase Cγ2 (Plcγ2), proline-rich tyrosine kinase 2 (Pyk2), and Src signaling pathways,^40^ which are essential for the resorbing activities.

During bone resorption, semaphorin 4D (Sema4D), an osteoclast-derived axon guidance molecule, suppresses bone formation on the surface by binding to Plexin-B1 on osteoblasts, activating the small guanosine triphosphatase (GTPase) ras homolog gene family, member A (RhoA), which inhibits insulin-like growth factor 1 (IGF-1) signaling and modulates osteoblast motility.^41^ Conversely, Sema3A produced by osteocytes and osteoblasts can inhibit ITAM-induced Plcγ2 activation and M-CSF-induced osteoclast differentiation through the RhoA signaling pathway and act as a potent osteoprotective factor.^42^ In addition to semaphorins, bidirectional crosstalk between osteoclasts and osteoblasts mediated by ephrin ligand-eph receptor (Ephrin-Eph) signaling and FAS ligand (FASL)-FAS signaling has also been emphasized during bone remodeling.^43^ Furthermore, resorption activity will trigger the release of coupling factors in the bone matrix, such as transforming growth factor-β (TGF-β) and IGF-1, which recruit osteoblast precursors (pOBs) to the surface and promote their differentiation as coupling factors.^44,45^ Collagen triple helix repeat containing 1 (CTHRC1) and S1P, secreted by active bone-resorbing osteoclasts, have also been found as coupling factors that promote osteogenesis by targeting stromal cells and S1P receptor 3 (S1PR3) on osteoblasts, respectively.^46^

Contrary to the default fate that bone-resorbing mOCs would exist for approximately two weeks and undergo apoptosis in previous dogma, recent studies have shown that the fission of mOCs into smaller daughter cells (osteomorphs) without resorbing abilities is a more common event than apoptosis,^47,48^ while these osteomorphs can migrate on the resorbing surface effectively and fuse into resorbing osteoclasts rapidly under RANKL stimuli at another site. Such recycling is more effective than apoptosis from a bioenergetic perspective and may be associated with denosumab’s side effects, which will be discussed below.

During osteoclast apoptosis, large amounts of apoptotic bodies containing nuclear components are secreted into the matrix. Among them, apoptotic bodies containing microRNA-214-3p (miR-214-3p) were demonstrated to suppress osteogenesis by binding osteoblast-specific transcription factors, such as Osterix and activating transcription factor 4 (ATF4), and promote osteoclastogenesis by decreasing phosphatase and tensin homolog (PTEN) through the phosphatidylinositol 3-kinase (PI3K)/Akt pathway (Fig. 1).^49–51^ Serum concentration of it in elderly women with fragile fractures and in ovariectomized (OVX) mice was also found to be increased,^52^ indicating its potential as a therapeutic target. Surprisingly, a reverse receptor-ligand signaling was recently found, wherein osteoclast-derived apoptotic bodies containing RANK could promote bone formation by binding to RANKL on osteoblast membrane, triggering the activation of Runt-related transcription factor 2 (Runx2, a crucial transcription factor regulating osteoblast proliferation and differentiation) by intracellular PI3K-Akt- mechanistic target of rapamycin kinase complex 1 (mTORC1) pathway,^53^ which may explain the transient decrease in bone formation observed in denosumab therapy.^54^ Nevertheless, the influence of these RANKs on osteogenesis is biphasic, considering their positive effect on early-stage osteoblast differentiation but a suppressive effect on late-stage differentiation of Runx2.^55^

### Osteocytes

As the most abundant (more than 90%) and long-lived (~25 years) cells embedded in the bone matrix, osteocytes regulate endocrine balance by controlling phosphate and insulin metabolism.^56^ In addition, osteocytes constitute an extensive three-dimensional (3D) network by interconnecting through dendrites. Through the 3D network, osteocytes detect mechanical cues by sensing fluid flow shear stress across their dendritic projections, thus adjusting the mechanical properties of bone and communicating with osteoblasts and osteoclasts via the RANKL/OPG axis and sclerostin/Dickkopf-1/WNT (SOST/Dkk1/WNT) axis. Previous evidence has demonstrated its significance in maintaining bone homeostasis, as age-related declines in the dendrite abundance and density can be observed and associated with a decreased lifespan of osteocytes, downregulated anabolic signals, and cortical fragility.^57^ A reduced individual osteon area and decreased dendrite canaliculi between osteocytes has also been observed in glucocorticoid-/glucose-related skeletal diseases with an obvious decline in connexin 43 (CX43) expression through the p38MAPK/ERK signaling pathway.^57,58^ Mechanistically, CX43 gap junctions account for intercellular communication, and CX43 hemichannels are responsible for signal and mediator exchange with the extracellular bone matrix, which is fundamental for the maintenance of dendrite network function.^59^ Impairment of CX43 expression induced by aging, estrogen deficiency, glucocorticoid treatment, and the high glucose microenvironment suppress cellular communication through the network and decrease osteocyte viability and bone turnover rate, eventually leading to the deterioration of the bone microstructure.^60–62^

Mechanical signals, such as oscillations of calcium ions, can be perceived by the osteocyte-bone lining cell syncytium located in the lacunocanalicular network of cavities filled with bone extracellular fluid, and induce the release of extracellular vesicles containing RANKL, OPG, sclerostin, and IGF-1 by osteocytes.^63^ Among these signaling molecules, sclerostin, a glycoprotein encoded by the SOST gene, plays a crucial role in the development of musculoskeletal system-related diseases by targeting SMAD1/5 to inhibit bone morphogenetic protein-2 (BMP-2)-induced osteogenesis or by competitively binding to the low-density lipoprotein receptor-related protein 5/6 (LRP5/6) coreceptor against WNT, thus triggering glycogen synthase kinase 3β (GSK3β)-mediated phosphorylation of β-catenin in the cytoplasm and form a complex to suppress its intranuclear translocation by ubiquitinated degradation thus decreasing WNT-related gene transcription (Fig. 5).^64^ Additionally, evidence has shown that sclerostin inhibits osteoblast differentiation by activating platelet-derived growth factor receptor (PDGFR) signaling,^65^ and PDGFR may act as a coreceptor in sclerostin-induced endocytosis of LRP6.^66^

During bone remodeling, osteocyte apoptosis is widely accepted as the initiating trigger of osteoclastogenesis and subsequent resorption. The apoptotic bodies from osteocytes were found to contain pro-osteoclastogenic factors such as RANKL, IL-6, intercellular cell adhesion molecule (ICAM)-1,^67^ high mobility group box 1 (HMGB1),^68^ and could upregulate sclerostin expression owing to increased mitochondrial uncoupling and superoxide production.^69^ An in vivo immunohistochemical analysis revealed a higher RANKL signaling in a 150–200 μm area around osteocytes undergoing apoptosis, whereas apoptosis inhibition resulted in a lower RANKL signaling.^70^ In contrast, apoptotic bodies of osteoblasts exhibit no impact on osteoclastogenesis either in vivo or in vitro.^71^ Apart from direct RANKL secretion by osteocyte apoptosis, evidence has also indicated that apoptotic osteocytes trigger RANKL production in healthy osteocytes nearby by activating the P2X7 and pannexin-1 receptors under ATP stimuli.^72^ Recent studies also suggested that damage-associated molecular patterns (DAMPs) derived from apoptotic osteocytes could trigger osteoclastogenesis through the ITAMs-based calcium signaling pathway by inducing macrophage-inducible C-type lectin (Mincle).^73^ Although there remain inconsistencies about whether osteoclastogenesis is activated directly or indirectly,^74^ it is clear that osteocyte apoptosis is associated with bone resorption and may serve as a therapeutic target. In fact, osteocytes provide the greatest source of RANKL during bone remodeling to promote osteoclastogenesis.^22^

### Osteoblasts

As osteoid-secreting cells on bone surfaces, osteoblasts account for 4–6% of total bone cells^75^ and originate from mesenchymal stem cells, undergoing pOBs and bone matrix-secreting osteoblasts, and eventually differentiate into bone lining cells and osteocytes. Fully differentiated osteoblasts are characterized by the coexpression of alkaline phosphatase (ALP) and type 1 collagen, which is essential for bone matrix and bone mineralization.^76^ Osteoblasts can also secrete RANKL, OPG, lysophosphatidic acid, and monocyte chemoattractant protein-1 (MCP-1) to regulate osteoclastic activities.^77^ WNT/β-catenin signaling plays a vital role in osteoblast activities and can be suppressed by extracellular and intracellular factors, such as sclerostin, Dkk1, secreted frizzled-related protein 1 (Sfrp1), and GSK3β.^78^ As osteoblasts mature, more inhibitory regulation on osteoclastogenesis occurs by releasing more OPG as the primary source during bone remodeling, which regulates the RANKL/RANK ratio to suppress bone resorption activities.^79^ However, similar to the osteogenic signal from osteoclasts, mature osteoblast-derived vesicles can induce a switch from bone formation to bone resorption by encapsulated RANKL and miR-143, a master regulator of osteoblastogenesis that inhibits Runx2 by targeting its dimerization partner, core-binding factor β.^80^

### Bone marrow mesenchymal stem cells

As the source of osteogenic-lineage cells, bone marrow mesenchymal stem cells (BMSCs) are distinguished by their self-renewal and multipotent differentiation capacities. During bone remodeling, WNT and correlative proteins-mediated osteogenic differentiation of BMSCs through the β-catenin-dependent (canonical) and β-catenin-independent (noncanonical) pathways is the initial step for bone formation. In particular, the activation of canonical WNT signaling suppresses mesenchymal stem cell differentiation to the chondrogenic and adipose lineages while promoting differentiation toward the osteoblastic lineage with increased OPG expression.^81,82^ At the end stage of bone resorption, a feedback loop for bone formation from osteoclasts could be observed by the secretion of WNT ligands such as WNT 10 and sphingosine 1 phosphate (S1P) (Fig. 5).^83^ In addition, T lymphocytes also express WNT-10b in bone marrow, which could promote bone formation and trigger osteoblast-derived signals on β-catenin through paracrine action.

Although late-stage β-catenin signaling has been found to possess a negative impact on osteogenesis,^84^ the upregulation of the WNT-β-catenin pathway generally leads to increased bone mass in most studies. Apart from classic WNT ligands such as WNT3a and WNT5b, and endogenous enhancers, including four R-spondin proteins,^85^ osthole (a coumarin derivative extracted from Cnidium monieri)^86^ and Foxf1 (Forkhead box protein f1) silencing^87^ were demonstrated to promote bone formation by activating the WNT-β-catenin pathway. In addition, bispecific WNT mimetics targeting Frizzled and low-density lipoprotein receptor-related proteins were designed by an antibody platform.^88^ Intraperitoneal injection of them generated swingeing and prompt bone formation effects in various murine models, including aging, osteoporosis, and fracture. Nevertheless, this osteogenic effect can be reversed by DKK-1, a soluble inhibitor that competitively binds to LRP5/6, triggering GSK3β complex mediated phosphorylation of β-catenin at the N-terminus and subsequent degradation of this key transcription factor.^89^ Hence, agents inhibiting the phosphorylation activity of GSK3β, such as indirubin-3’-oxime (I3O)^90^ and MK2206^91^ (Fig. 7c), can also be used to promote osteogenic differentiation of BMSCs with verified preclinical effects. Nevertheless, the broad distribution of β-catenin signaling in other tissues and the promotion of arthritis are potential negative consequences to overcome.^92,93^

### Immune cells

**Fig. 2:**
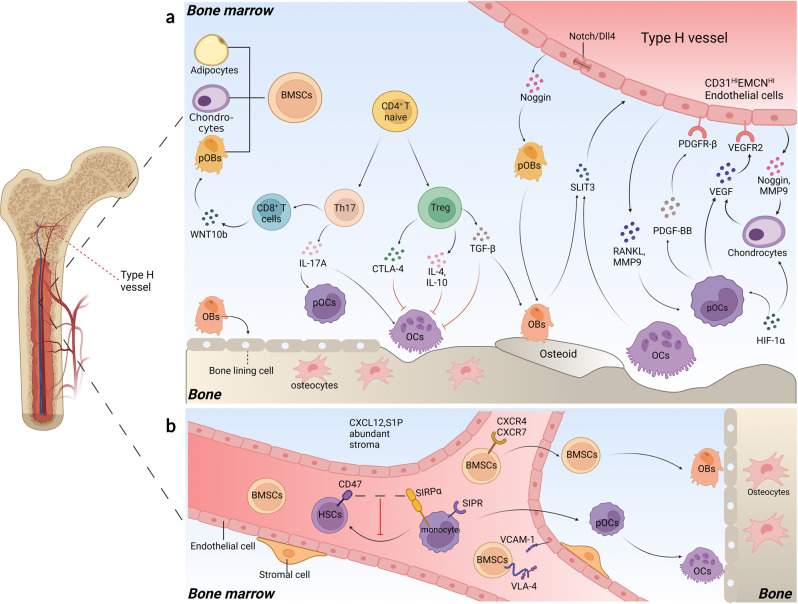
Angiogenesis, homing, and the immune microenvironment in the bone marrow. **a** Th17 cells promote osteoclast differentiation by secreting IL-17A to upregulate RANKL, TNF-α, and IL-6, while Treg cells suppress osteogenesis by secreting inhibitory cytokines such as TGF-β, IL-4, and IL-10 and enhance WNT-10b expression via interaction with CD8^+^ T cells. CTLA-4 expressed by Treg cells can degrade tryptophan and promote pOC apoptosis by binding to CD80/86 on the surface of pOCs. SLIT3, PGDF-BB, and VEGF secreted by osteoblasts, osteoclasts, pOCs, and chondrocytes can promote type H angiogenesis. Endothelial Notch/Dll4 signaling can increase Noggin secretion from type H endothelial cells (ECs), which promotes osteogenesis and chondrocyte hypertrophy maturation. RANKL and MMP9 derived from type H ECs can facilitate osteoclast chemotaxis and osteoclastogenesis. **b** CXCR4/7, integrin α4β1 (VLA-4), and S1PR can respond to CXCL12, VCAM-1, and S1P to mediate the homing of BMSCs and pOCs. CD47 on hematopoietic stem cells (HSCs) serves as a ‘marker of self’ that binds to CD172α (S1RPα) on phagocytes to reduce depletion from mononuclear phagocyte system during homing

Over the past few decades, attention has been given to the role of bone marrow immune cells in regulating bone remodeling.^94^ Sjögren et al. first reported the phenomenon that germ-free mice, which were distinguished by a decrease in CD4^+^ T cells and CD11b^+^/GR 1^−^ pOCs in the bone marrow, and lower levels of inflammatory cytokines, such as interleukin- 6 (IL-6) and tumor necrosis factor α (TNF-α), tend to exhibit increased bone mass and decreased osteoclast number.^95^ Subsequent studies validated the role of the Treg/Th17 axis during bone marrow bone remodeling. Mechanistically, Th17 cells promote osteoclastogenesis by producing IL-17A, which serves as a receptor activator of RANKL, TNF-α, and IL-6.^96^ In contrast, Treg cells suppress osteoclastogenesis by secreting inhibitory cytokines, such as TGF-β, IL-4, and IL-10,^97^ and enhance WNT-10b expression via interaction with CD8^+^ T cells (Fig. 2a).^98^ CTLA-4 expressed by Treg cells can also bind to CD80/86 on the surface of pOCs and activate indoleamine-2,3-dioxygenase (IDO), which can degrade tryptophan and promote pOC apoptosis.^99^ Treg cells can also directly promote osteogenic proliferation and differentiation of BMSCs by secreting TGF-β, which activates intracellular regulators such as MAPK and SMAD-related proteins.^100^ Notably, a higher proportion of activated Th17 cells has been observed in postmenopausal women due to a lack of estrogen suppression of Th17 cell-derived inflammatory cytokines.^101^

A recent study showed that Lactobacillus rhamnosus (LR, a type of probiotic) supplementation effectively restored bone loss in OVX mice, accompanied by an improved Treg/Th17 balance in bone marrow, Peyer’s patches, spleen, and lymph nodes.^15^ Although the underlying mechanism remains elusive, it has been hypothesized that microbial metabolites and immune regulation may be implicated via the gut-bone axis.^102^ In addition, T-cell depleting nanoparticles (NPs), consisting of an MCP-1-encapsulated mesoporous silica core and FASL corona, have been found to ameliorate bone loss by suppressing activated T cells and regulating the Treg/Th17 balance.^103^ In particular, the released MCP-1 facilitates the recruitment of activated T cells and triggers their apoptosis by FASL on the surface, which can be recognized by macrophages, thus reversing the Th17/Treg ratio in the bone marrow immune microenvironment. These remarkable results not only underscore the significance of cellular crosstalk during bone remodeling but also indicate the feasibility of immune cell-targeted therapies for promoting bone homeostasis.

### Type H vessel endothelial cells

As a critical portion of the bone marrow environment, the blood vasculature has garnered attention in recent studies for its role in bone remodeling.^104^ Based on marker expression and functions of endothelial cells (ECs), type H and type L vessels are distinguished.^105^ Located in the bone marrow cavity of the diaphysis, type L vessels form a highly branched and dense capillary network and are surrounded by leptin receptor (LEPR)^+^ and CXCL12-rich reticular (CAR) perivascular cells, which are known for their roles in stem cell homing and hematopoiesis.^106–108^ In contrast, type H vessels exhibit a more significant role in bone homeostasis regulation with Osterix^+^ and Runx2^+^ osteoprogenitors and collagen type 1α^+^ osteoblasts surrounded, which do not exist near type L vessels.^105,109^ The expression of bone formation transcripts, such as platelet-derived growth factor (PDGF) A/B, transforming growth factor (TGF) β1/β3, and fibroblast growth factor 1 (FGF1), is also prominently higher in type H vessel cells (ECs) than in type L ECs.^105^ In addition, a decrease in type H vessels and osteoprogenitors can be observed in aged, OVX, and diabetic osteoporosis (DOP) mice, while the total number of endothelial cells remains unchanged due to the increase in type L vessels.^14,105,110^

Generally, type H vessels are located near the growth plate in the metaphysis and periosteum of the diaphysis with high expression of CD31 and endomucin (CD31^HI^EMCN^HI^) (Fig. 2). Recent studies have revealed the crosstalk between type H ECs and bone/cartilage cells during bone/cartilage remodeling (Fig. 2a).^105,111^ pOC-derived platelet-derived growth factor type BB (PDGF-BB) is secreted into the periosteum and recruits periosteal progenitor cells for endothelial and osteogenic progenitor cell differentiation, leading to a coupling of type H angiogenesis and periosteal bone formation.^14^ Harmine, a β-carboline alkaloid, has been shown to enhance type H vessel formation and reverse bone loss in OVX mice by promoting pOC-derived PDGF-BB.^112^ In addition, slit guidance ligand 3 (SLIT3), a Schnurri-3-regulated proangiogenic factor secreted by mature osteoblasts and osteoclasts, can facilitate endothelial tube formation and the branching of type H vessels.^113^ Administration of recombinant SLIT3 or deletion of Schnurri-3 (Shn3) reversed the bone loss in OVX mice with enhanced expression of CD31^HI^EMCN^HI^ endothelium. As transducers of intercellular signaling between ECs, Notch and its ligand delta-like 4 (Dll4) are also associated with bone formation.^111^ Upregulation of Notch/Dll4 was demonstrated to increase Noggin secretion from type H ECs, which promotes perivascular osteoprogenitor cell differentiation, chondrocyte hypertrophy maturation, and EC proliferation. Type H ECs can mediate cartilage resorption and longitudinal bone growth by secreting RANKL and MMP9 to regulate osteoclastogenesis and osteoclast migration.^114^ Other factors, including hypoxia-inducible factor 1-alpha (HIF-1α) and vascular endothelial growth factors (VEGFs), derived from chondrocytes, osteoblasts, and pOCs have been discovered in the coupling of osteogenesis and angiogenesis.^115^ Zhuang et al. found that small extracellular vesicles (EVs) derived from hypoxic MSCs could overexpress miR-210-3p under HIF-1α inducing, and such hypoxia-preconditioned MSC-derived EVs (hypo-sEVs) significantly enhanced CD31^HI^EMCN^HI^ type H vessel vascularized bone regeneration through the miR-210-3P/EFNA3/PI3K/Akt pathway in a calvarial bone repair rat model.^116^

These findings underscore the significance of type H vessels in bone remodeling and provide potential targets for bone-related diseases. Nevertheless, excessive type H vascularization in the subchondral bone has also been demonstrated to promote arthritis progression.^117^ A recent study revealed that PDGF-BB could promote the occurrence of osteoarthritis (OA) by enhancing angiogenesis-dependent abnormal subchondral bone formation through the PDGFR-β/talin1/FAK pathway, whereas PDGFR-β deletion or local injection of adeno-associated virus serotype 9 (AAV9) carrying PDGFR-β shRNA in subchondral bone reversed the progression in OA models.^118^ How to promote bone formation without dysregulating the subchondral bone microenvironment may be a potential obstacle for therapies targeting type H vessels.

## Drug targets based on remodeling biology

**Fig. 3:**
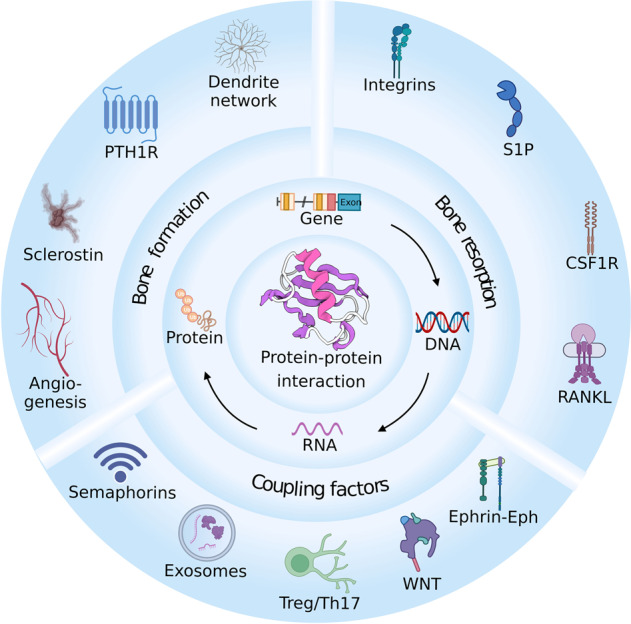
Therapeutic targets for improving bone homeostasis. Intercellular activities are mediated by specific protein–protein interactions (PPIs). By targeting key gene or protein expression, PPIs during bone remodeling can be regulated to improve bone formation and alleviate bone resorption

With a deeper comprehension of bone remodeling biology, the mechanism underlying the side effects of some established drug targets has surfaced with potential improvements. Other druggable targets have also been exploited in preclinical studies with promising therapeutic potential (Fig. 3).

### Established drug targets

#### Receptor activator for nuclear factor-κB ligand (RANKL)

The necessity of RANKL for osteoclastogenesis has led to the development and approval of denosumab, a human monoclonal antibody against RANKL. A 12-month trial comparing denosumab and alendronate in postmenopausal osteoporosis revealed that denosumab was more effective in bone mineral density (BMD) improvement and cortical porosity decrease, suggesting that denosumab is a better short-term option noticing its more convenient administration model and more rapid effect.^119^ However, concerns were raised regarding its rebound osteoclast activity and the risk of multiple spontaneous vertebral fractures after withdrawal.^120^

The higher rebound osteoclast activity of denosumab after withdrawal compared with bisphosphonates (BPs) may result from their different anti-resorption mechanism. Nitrogen-containing BPs inhibit the activity of farnesyl diphosphate synthase (FDPS), which blocks the prenylation of small GTPases (such as Ras, Rho, and Rac) that are necessary signaling mediators for maintaining the cytoskeleton and forming fold edges, thus suppressing osteoclast activity and promoting its apoptosis.^121^ Nonnitrogen-containing BPs are metabolized into ATP analogs with methylene in cells on the bone surface, which causes cytotoxicity by the nonhydrolyzed P-C-P structure. Compared with the apoptosis process that requires high energy for apoptotic debris removal, denosumab treatment inhibits osteoclastogenesis and the refusion of non-resorbing osteomorphs into osteoclasts, which is a more effective process in terms of energetics (Fig. 5).^47^ However, with the accumulation of osteomorphs, upon administration suspension, a high dose of RANKL exposure would quickly revert these silenced cells into active bone-resorbing osteoclasts, thus generating a massive bone loss in a rapid time,^122^ and sequential BP treatment is often conscripted to prevent fractures.^123^

To alleviate such rebound osteoclast activity, efforts have been made to target LGR4, another RANKL receptor, against RANK. In Luo et al.’s study, the soluble LGR4 extracellular domain (LGR4-ECD) was demonstrated to bind RANKL and reverse excessive RANKL-induced bone loss in OVX mice. Notably, little effect on physiological osteoclast differentiation in normal mice was caused by LGR4-ECD, probably due to its lower affinity with RANKL than endogenous OPG, which indicates that it may serve as an antagonist of excessive RANKL in pathological conditions with less rebound resorption risk compared to denosumab or OPG agents.^32^ In addition, a modified RANKL sequence with changes of five amino acids in the binding site that acts as an inhibitory RANKL vaccine has recently been developed to specifically bind to LGR4.^124^ Activated LGR4 suppressed NFATc1 expression through the GSK3β pathway (Fig. 1). Surprisingly, it could also trigger the generation of RANKL-specific antibodies, probably due to residue effects. Although further validation is required to determine whether it can decrease side effects such as rebound resorption risk and calcium homeostasis imbalance, these results indicate that LGR4 could be a promising target for regulating osteoclast resorption, noticing its lower expression on pOCs than mOCs and less influence on physiological osteoclastogenesis.

Apart from the risk of rebound resorption, concerns regarding the latent immunosuppressive effects of denosumab have also been raised since mRANKL is also expressed as a type 2 transmembrane protein belonging to the tumor necrosis factor superfamily on immune cells.^125^ Thus, the usage of RANKL antibodies may disrupt the reverse RANK-RANKL signaling pathway, which mediates normal immune processes, such as cell proliferation, survival, and thymus and lymph node development.^126,127^ In view of this, a reformative strategy was proposed to target sRANKL, which lacks a C-terminal extracellular connecting stalk domain and does not participate in the reverse RANK-mRANKL signaling in immune cells.^128^ Although osteoclastogenesis is mainly promoted by mRANKL from osteogenic cells,^22^ recent studies have also confirmed sRANKL-mediated segmental osteoclastogenesis and bone resorption during bone remodeling.^129,130^ In Huang et al.’s investigation, S3-15 was screened through molecular dynamics studies as a potent inhibitor targeting the binding of mouse sRANKL to RANK.^131^ In vivo and in vitro studies demonstrated an anti-osteoporosis effect without accompanying immunosuppression, which validated its specific targeting of the specific protein–protein interactions (PPIs) between sRANKL and RANK, thus offering a potential avenue for developing novel RANKL inhibitors.

#### Sclerostin

Recent evidence has established a compelling correlation between high serum sclerostin levels and postmenopausal osteoporosis-related fractures.^132^ As a potent suppressor of bone formation, sclerostin has become an attractive target in anabolic bone therapies. Romosozumab (AMG785), the pioneer sclerostin inhibitor with FDA approval, has demonstrated a tremendous therapeutic effect in postmenopausal osteoporosis.^133^ Several other promising anti-sclerostin antibodies, including BPS804 (setrusumab)^134^ and SHR-1222,^135^ have also emerged on the horizon in trials of osteoporosis or osteogenesis imperfecta treatment. Mechanistically, these sclerostin antibodies (Scl-abs) function by moderating the binding of sclerostin to LRP5/6 to increase β-catenin concentration and decrease the negative suppression of WNT-induced responses.^136^

In a 12-month randomized controlled study (RCT) comparing the efficiency of alendronate and romosozumab, Saag et al. found that the romosozumab injection group exhibited a 48% lower risk of new vertebral fractures.^137^ Despite the superiority in reducing fracture risk, romosozumab treatment is associated with adverse drug reactions, including arthralgia, headache, peripheral edema, and severe cardiovascular events such as stroke and heart attack, which have hindered its further application.^138^ A meta-analysis of 25 cardiac events in 4298 individuals from two phase 3 randomized controlled trials of romosozumab further validated its higher risk of cardiovascular events at a dose of 210 mg per month (odds ratio = 2.98, 95% CI: 1.18–7.55, *P* = 0.02).^139^ Moreover, the study also showed that BMD-increasing SOST variants (rs7209826 (G-allele) and rs188810925 (A-allele)) were associated with a lower sclerostin expression and a higher cardiovascular risk,^139^ indicating that both pharmacological inhibition by sclerostin antibodies and SOST gene defects can lead to an elevated risk of cardiovascular events.

**Fig. 4:**
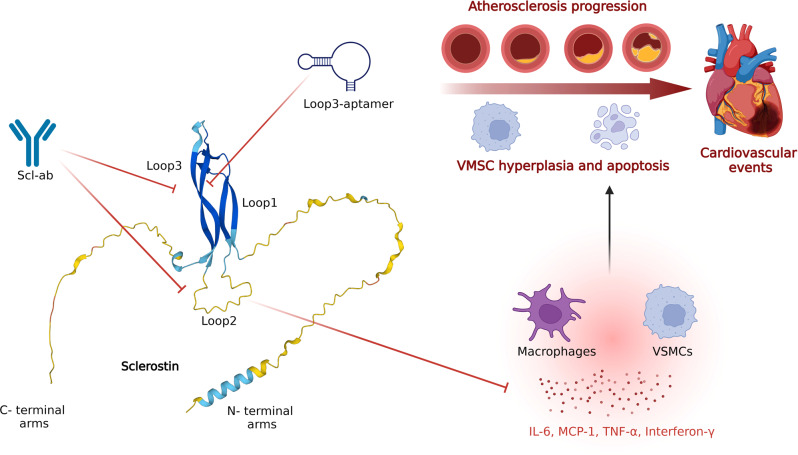
Loop2 and loop3 of sclerostin are key binding targets for sclerostin antibodies (Scl-abs). Both of them can mediate the bone formation suppression effect of sclerostin, while loop2 possesses a cardiovascular protective effect by decreasing inflammatory cytokines and chemokines such as IL-6, MCP-1, TNF-α, interferon-γ, et al., in VSMCs and macrophages. Scl-abs inhibit the functions of both loop2 and loop3, thus promoting bone formation but increasing cardiovascular risk. In contrast, the loop3-aptamer inhibits sclerostin’s bone suppression effect while preserving the cardioprotective effect of loop2

Although the cardiovascular risk has been well-recognized since the approval of romosozumab, no effective measures were identified until a recent report by Yu and Wang et al. that targeting loop3 of sclerostin may attenuate cardiovascular risk while retaining its skeletal protection.^140^ Structurally, sclerostin is composed of a core cystine knot structure of three loops (loop1, loop2, and loop3) and long, highly flexible, and unstructured N- and C- terminal arms (Fig. 4).^141^ Loop1 and loop3 form a structured β-sheet, while loop2 is unstructured and highly flexible.^142^ It was previously revealed that loop2 and loop3 of sclerostin are two main binding sites for Scl-abs,^141^ and loop2 is critical for sclerostin’s WNT inhibition,^142^ which makes the identification of the roles of these loops in the cardiovascular risk of Scl-abs worthwhile.

In Yu et al.’s study, loop3-deficient sclerostin knock-in mice exhibited similar cardiovascular protection in apolipoprotein E deficient (ApoE^−/−^) mice with angiotensin II (AngII) infusion as the full-length sclerostin knock-in (hSOST^ki^) mice did, while attenuating the inhibitory effect on bone formation with a similar bone parameter to the wild-type groups.^143^ The expression of WNT signaling and osteogenic markers such as osteocalcin (OCN) and ALP were also significantly higher in loop2 and loop3-, and loop3-deficient MC3T3-E1 cells than in cells with full-length sclerostin. In addition, exogenous loop2 supplementation reversed Scl-ab-induced increased cardiovascular risk with better aortic parameters and less immune cell infiltration, cell apoptosis, and contractile phenotype loss of aortic vascular smooth muscle cells (VSMCs). These results indicate that both loop2 and loop3 participate in sclerostin’s inhibition of bone formation, while loop2, rather than loop3, is responsible for sclerostin’s cardiovascular protection. Thus, targeting loop3 could be a promising therapeutic avenue with decreased concerns of cardiovascular events.

Mechanistically, the cardiovascular risk of Scl-abs stems from their suppression of loop2, which mediates sclerostin’s inhibitory effect on inflammatory cytokines and chemokines such as IL-6, MCP-1, TNF-α, interferon-γ, et al., in VSMCs and macrophages, thus preventing the genesis of abdominal aortic aneurysm and atherosclerosis (Fig. 4).^144,145^ Thus, targeting loop3 of sclerostin can partially reverse sclerostin’s bone inhibition without affecting loop2’s cardiovascular protection. In Yu et al.’s study, an aptamer aptscl56 was identified to specifically target loop3, and its modified version, APC001PE (PEG40K-aptscl56), interdicted the antagonistic effect of sclerostin on WNT signaling in bone with cardiovascular protective effects retained in vitro. It was demonstrated to improve bone formation in hSOST^ki^ mice, OVX, and osteogenesis imperfecta mice without affecting aortic aneurysm and atherosclerotic development and proved nontoxic to healthy rodents even at an ultrahigh dose.^143,144^ Due to these efficacies, it was granted orphan drug designation for osteogenesis imperfecta by the FDA in 2019 (DRU-2019-6966), which may pioneer a novel target for developing next-generation sclerostin inhibitors.

Of note, irisin, an endogenous mediator secreted by the muscle in response to physical activities, has been found to prevent disuse-induced osteocyte apoptosis and upregulate sclerostin expression by targeting its main receptor integrin α5β5 on osteocytes through the ERK/ATF4 signaling pathway.^146,147^ In particular, exogenous irisin supplementation increased bone resorption by promoting SOST expression, while genetic ablation of irisin or its precursor protein fibronectin type III domain-containing protein 5 (FNDC5) in muscle-blocked osteocytic osteolysis in OVX mice.^146^ Nevertheless, low-dose intermittent injection of irisin has been reported to improve cortical bone mineral density and strength in mice by decreasing SOST expression in osteocytes and activating BMP/SMAD signaling in BMSCs,^148,149^ resembling the action of parathyroid hormone (PTH). In addition, although FNDC5-knockout caused lower RANKL mRNA expression and improved femoral trabecular bone mass and connectivity density in female mice, no bone structure change was observed in male mice, and the OPG level was not altered in either male or female mice.^146^ These different results revealed the intricate regulatory mechanism of irisin in bone homeostasis, and it is also elusive whether the main skeletal effect is mediated by osteocytes. Further research is required to elucidate the mechanism for developing irisin-based anti-sclerostin therapy, which is worthwhile considering its cardiovascular benefit.^150^

#### Type 1 parathyroid hormone receptor (PTH1R)

As the primary receptor for endogenous PTH and PTH-related peptides (PTHrP), PTH1R, a class B G protein coupled seven transmembrane receptor, plays a puissant role in regulating calcium/phosphorus metabolism and bone homeostasis. Teriparatide, a bioactive N-terminal segment of PTH residues 1–34, and abaloparatide, an analog of PTHrP, were synthesized and approved by the FDA as long-acting and short-acting peptides to treat osteoporosis, respectively.^5^ Notably, the effect of PTH1R activation on bone metabolism is twofold: the anabolic effect relies on intermittent dosing, and the catabolic effect is attained at consistently high dosing.^151^ To realize the anabolic effect, a 20 μg/day dose of teriparatide and an 80 μg/day dose of abaloparatide subcutaneously for 18–24 months are recommended^5^ for an appropriate exposure duration.^151^ Although the short-term anabolic efficacy of teriparatide is superior to that of BPs, it was bothered by frequent injections, hypercalcemia, and risk of osteosarcoma and has a restricted treatment duration of approximately 2 years due to safety concerns.^5^

Mechanistically, the binding of teriparatide and abaloparatide to PTH1R activates multiple signaling pathways, including Gs/PKA/cAMP, Gq/phospholipase C/Ca^2+^, and β-arrestin/ERK pathways, to trigger the expression of anabolic genes.^152^ Among them, stimulatory G protein (Gs) signaling is considered the primary mediator of bone and calcium regulation.^153^ In a study by Nemec et al., receptor-activity-modifying protein 2 (RAMP2) was proven as a specific allosteric modulator of PTH1R that increases PTH’s selective activation of Gs and Gi3 signaling and increases β-arrestin2 recruitment to PTH1R triggered by PTH and PTHrP.^154^ It also promoted a faster activation of PTH1R by these ligands and reduces their activating amplitude, which may decrease the potential catabolic effect.^151^ Although the mechanism of RAMP2-induced binding features and downstream interaction alterations is still elusive, it offers the possibility of developing adjunct drugs for PTH1R ligands, as RAMP2 itself cannot activate G proteins.

Notably, a more pronounced effect on PTH in comparison to PTHrP by RAMP2 was observed in the study,^154^ which may be attributed to the distinct activation features of teriparatide and abaloparatide on PTH1R. Despite the highly similar interactions downstream of PTH1R mediated by PTH and PTHrP, the stability and signaling duration of PTH1R activation vary significantly. PTHrP induces a more rapid dissociation from PTH1R and faster cAMP decay than PTH,^152^ which may be associated with the R_G_/R_0_ conformations of PTH1R. PTH and long-acting teriparatide possess a similar binding affinity to both R_G_ and R_0_ conformations with a 2- to 10-fold difference,^155^ whereas the short-acting abaloparatide possesses a similar R_G_ affinity compared to PTH, but a 100- to 1000-fold lower affinity for R_0_.^156^ Recent 3D variability analysis and site-directed mutagenesis studies based on cryo-electron microscopy have further identified the critical residue (I/H in position 5) that differentiates affinities with the R_0_ state receptor R.^157^ High affinity to both the R_0_ and R_G_ states maintain multiple cycles of G protein coupling and dissociation, leading to a sustained duration time for PTH and teriparatide. In contrast, transient signaling is maintained by abaloparatide due to its unstable R_G_ state.^158^ These results provide potent evidence to support the assumption that the duration of Gs-mediated cAMP production can modulate the balance between anabolic and catabolic effects since less bone resorption and hypercalcemia have been observed by PTHrP than by PTH.^159^ Although the specific mechanism requires further investigation, these results reveal potential targets for improving PTH1R ligands.

### Potential druggable targets

#### Membrane expression

##### Colony-stimulating factor 1 receptor (CSF1R)

CSF1R is a type 3 receptor tyrosine kinase that plays an essential role in the genesis and maturation of myeloid cells, including pOCs. Upon binding with M-CSF and IL-34, CSF1R undergoes tyrosine phosphorylation, of which Tyr559 and Tyr807 phosphorylation are essential for PI3K-Akt signaling-mediated osteoclastogenesis, and Tyr697 phosphorylation is potent for integrin β3 expression and GRB2/ERK signaling, promoting cell proliferation and survival (Fig. 1).^160^ In addition, CSF1R-mediated signaling is essential for the expression of RANK.^161^ The role of the M-CSF/CSF1R axis has been well established in M1 macrophage polarization in rheumatoid arthritis,^162^ while selective deletion of the soluble CSF1 isoform or using CSF1R antibodies has also been validated to improve bone mass.^163,164^ Recently, a multikinase inhibitor, YKL-05–099 (Fig. 7c),^165^ was proven to be a promising anabolic agent without the potential risk of osteolysis targeting both CSF1R and salt inducible kinases (SIKs), the latter being broadly expressed AMPK family serine/threonine kinases regulated by cAMP signaling and can be suppressed by PTH by mediating the phosphorylation of SIK2 and SIK3.^166^ In addition, a novel bispecific inhibitor of CSF1R and α5β3-integrin has been developed by replacing one of the two loops on the M-CSFC31S (a mutant M-CSF with cysteine in position 31 thus transforming from an agonist to an antagonist of the CSF1R) scaffold with RGD,^167^ suppressing CSF1R- and α5β3-mediated osteoclastogenesis and bone resorption processes simultaneously. The success of the M-CSF_RGD_ variant leaves a vast stage in synthesizing drugs with multiple targeting sites during osteoclastogenesis and osteoclast activities.

##### Integrins

Integrins are the main cell-adhesion transmembrane molecules in vivo and have recently been considered potential drug targets for multiple biological events.^168^ As an essential molecule in the resorption lacuna, α5β3 has raised interest as a novel anti-resorption target since its discovery in the 1980s.^169^ Early works using α5β3 antibodies or competitive ligands verified its effect in vitro.^170,171^ α5β3 antagonist supplementation also reversed bone loss and improved BMD in postmenopausal women with osteoporosis.^172^ Recently, using in silico docking method-integrated protein chip technology, Park et al. also screened a novel small molecule inhibitor targeting α5β3, IPS-02001 (Fig. 7c), with a verified effect in OVX mice.^173^ However, it has been shown that α5β3 is the unique integrin on BMSCs that mediates their endocytosis of primary and circulating apoptotic bodies, reusing apoptotic body-derived ubiquitin ligase RNF146 and miR-328-3p, which can inhibit Axin1 and activate the WNT/β-catenin pathway.^174^ Therefore, further research is required to exploit the influence of α5β3 inhibition on bone remodeling rather than onefold bone resorption.

Intriguingly, α5β5 integrins on pOCs, which recognize the same amino acid motif as α5β3, were found to negatively regulate osteoclastogenesis.^175^ β5 deletions in mice showed enhanced osteoclastogenesis and resorption activity under estrogen deficiency. It was also proven as a receptor of irisin, a muscle-derived bone regulator on bone cells.^146^ In addition, integrins with osteogenic effects have also been discovered. α5β1-integrin enhances osteoblastic differentiation of MSCs through the FAK/ERK signaling pathway and suppresses osteogenic cell apoptosis through the FAK/PI3K/Akt survival pathway.^176^ It also participates in the local release of bone anabolic molecules such as prostaglandins through the CX43 hemichannel under mechanical loading by the PI3K/Akt pathway.^177^ Decreased α5β1-integrin expression was detected in unloading rats, while exogenous activation of α5β1 led to increased bone formation and improved bone repair in mice. Integrin α4β1 expressed on BMSCs responds to chemokines CXCL12 (C-X-C motif chemokine ligand 12, also known as stromal cell-derived factor 1, SDF-1) and vascular cell adhesion molecule-1 (VCAM-1) to promote BMSC homing,^178^ which benefits bone formation. These studies indicated that integrins could be therapeutic targets for bone diseases. Nevertheless, due to its extensive biological participation, promoting tumor metastasis and other potential side effects should be considered.^168^

##### Sphingosine 1 phosphate receptor (S1PR)

Recruitment of circulating pOCs to bone marrow and their differentiation into osteoclasts are crucial processes for bone resorption. The inhibition of intermediate molecules that mediate the homing and fusion of pOCs has emerged as a promising target for anti-resorption therapy. The fusion of pOCs into multinucleated mOCs requires circulating pOCs outside bone marrow to be recruited to the remodeling site, which is co-mediated by stroma-derived factor 1 (SDF-1) produced by bone marrow cells and S1P secreted by red blood cells and platelets in the circulation.^179,180^ The response of pOCs to SDF-1 and S1P signaling is mediated by S1P receptors 1 and 2 (S1PR1,2) on the pOC membrane. S1PR1 signaling chemoattracts pOCs from the marrow to the blood, whereas S1PR2 chemorepels them back to the marrow niche.^181^ In addition, previous studies found higher levels of resorption-related markers, such as α5β3, RANK, cathepsin K, and MMP9, but lower levels of SIPR, on large differentiated mOCs (>10 nuclei) than in small mOCs, indicating its fusion-mediating ability.^182^ Furthermore, as mentioned above, S1PR3 on osteoblasts can receive mOC-derived coupling signals to stimulate osteogenesis.^181^ Administration of FTY-720 (Fig. 7c), a nonspecific S1PR1 agonist, was also demonstrated to reverse bone loss in OVX mice.^183^ These results indicate the potential to target S1PR for therapeutic interventions.

#### Cellular crosstalk

##### Semaphorins

**Fig. 5:**
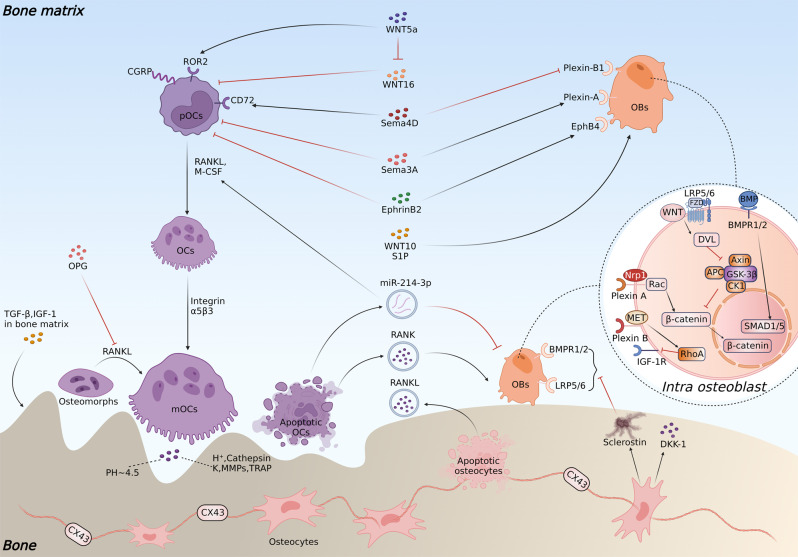
Cellular crosstalk among osteoblasts, osteoclasts, and osteocytes during the remodeling process. Osteoblast- and osteocyte-derived OPG can suppress the fusion of osteomorphs into osteoclasts. Sema4D promotes osteoclastic resorption by binding CD72 on pOCs, while Sema3A, produced by osteocytes and osteoblasts, inhibits osteoclastogenesis. WNT5a expressed by osteoblasts can stimulate the differentiation of pOCs in the noncanonical pathway by binding to ROR2 and reversing the inhibitory effects of WNT16 on RANKL-induced osteoclastogenesis. EphrinB2 secreted by osteoclasts can bind to EphB4 on osteoblasts and promote osteogenic differentiation by inhibiting the small GTPase RhoA, whereas reverse Eph signaling on pOCs can inhibit osteoclastogenesis by downregulating c-FOS and NFATc1 expression. Apoptotic osteoclasts secrete miR-214-3p, which suppresses osteoblast-specific transcription factors such as Osterix and ATF4 and promotes osteoclastogenesis by decreasing PTEN through the PI3K/Akt pathway. Conversely, RANK secreted by apoptotic osteoclasts can activate Runt-related transcription factor 2 and the intracellular PI3K-Akt-mTORC1 pathway. Sclerostin secreted by osteocytes can inhibit osteogenesis by binding to the LRP5/6 coreceptor to promote GSK3β complex-mediated inhibition of anabolic β-catenin signaling and inhibiting BMP-2/SMAD1/5-induced osteogenesis. Osteocyte apoptosis is accompanied by the secretion of RANKL, which promotes the resorption process. Sema4D inhibits IGF-1-mediated osteoblastic formation by binding the Plexin-B1 receptor expressed on osteoblasts. Sema3A acts on Plexin-A and neuropilin 1 (Nrp1) on pOBs to promote osteogenesis through the Rac signaling pathway

Recently, semaphorin-mediated crosstalk between osteoclasts and osteoblasts has been considered a potent coupling signal during bone remodeling. Apart from bone formation suppression by the Plexin-B1/IGF-1 signaling pathway,^41^ Sema4D also promotes osteoclastic resorption and osteoclastogenesis by binding CD72 on pOCs.^184^ Systemic administration of Sema4D-specific siRNAs enhanced bone formation and decreased bone resorption in healthy and OVX mice.^41,185^ Implantation of scaffolds loaded with Sema4D siRNA also reversed bone defects in mouse models.^186^ In addition, PB1m6, a macrocyclic peptide with high Plexin-B1-specific affinity, was synthesized to target the Sema4D/Plexin-B1 interaction, which is difficult for traditional small-molecule drugs due to the large and flat binding surface of the semaphorin-Plexin interaction interface.^187^ Conversely, Sema3A was discovered as a significant osteogenic coupling messenger by inhibiting Plcγ2- and M-CSF-induced osteoclast differentiation and promoting Plexin-A- and neuropilin 1 (Nrp1)-induced osteogenesis via the canonical WNT/β-catenin signaling (Fig. 5). It was demonstrated that such osteogenic effect could be promoted by estrogen through suppressing Sema3A-inhibiting miRNAs.^188^ Fukuda et al. further proved that both Sema3A in bone and from the nervous system are involved in bone homeostasis regulation, and Sema3A possesses an additional osteogenic effect by modulating sensory nerve development in bone in addition to the direct effect induced by targeting osteocytes.^189^ Systemic administration of Sema3A-specific agonists also enhanced bone formation and inhibited bone resorption in healthy and OVX mice.^190^ These dual regulatory effects of semaphorins in bone remodeling are exciting, considering that previous antiresorptive therapies tend to slightly inhibit bone formation while anabolic treatments would also increase bone resorption to a small extent.^7^

Intriguingly, in an evaluation of circulating Sema4D and Plexin-B1 levels in postmenopausal women with low bone mass after 3 months of treatment with zoledronic acid, denosumab, and teriparatide, Sema4D levels were not significantly affected by zoledronic acid but were increased by denosumab and decreased by teriparatide.^191^ The distinction between denosumab and zoledronic acid could probably result from their different action models or the unique effect of denosumab on lymphocyte-derived receptor activator of RANKL and the RANKL-mediated immune response, as T cells are also known producers of Sema4D.^192^ Although it was a short-term study, it indicated that anti-resorption therapy may lead to higher Sema4D expression, which can be a risk factor for the reactivation of bone resorption after withdrawal. Thus, an ancillary anti-Sema4D moiety may foster better anti-resorption therapy by preventing Sema4D accumulation.

##### Ephrin-Eph signaling

In addition to semaphorins, Ephrin-Eph signaling-mediated cellular crosstalk has recently been emphasized in bone homeostasis.^193^ EphrinB2, a transmembrane protein with cytoplasmic domains on osteoclasts, can bind with the receptor EphB4 on osteoblast membranes and promote osteogenic differentiation by inhibiting the small GTPase, RhoA.^194^ In contrast, reverse EphrinB2 signaling on pOCs can inhibit osteoclastogenesis by downregulating c-FOS and NFATc1 expression.^194^ Previous studies also showed that EphrinB2 signaling was required for the PTH-mediated anabolic effect.^195,196^ Furthermore, EphA4 has been identified as another negative regulator of osteoclast activity.^197^ In particular, EphA4 inhibits β3-integrin signaling by increasing phosphorylation of the Tyr-747 residue, leading to decreased binding of the stimulatory talin and increased binding of the suppressive docking protein 1 (Dok1) to β3-integrin. EphA4 deletion led to more giant osteoclasts with higher expression of MMP3 and MMP9, while activation by EphrinA4-fc chimeric protein suppressed bone resorption by activating EphA4.^197^ In addition, selective delivery of miR-141 was proven to inhibit excessive bone resorption in aged rhesus monkeys by targeting calcitonin receptors and EphA2.^198^ These findings underscore the therapeutic potential of Ephrin-Eph signaling in bone homeostasis.

##### Extracellular vesicles

As phospholipid bilayer-enclosed vesicles secreted by all cells, extracellular vesicles (EVs) have been considered significant messengers in cellular crosstalk and biological activities. Generally, EVs can be classified into three categories (exosomes, apoptotic bodies, and micro-vesicles) based on their biogenesis and size.^199^ A diverse range of cargos have been identified in bone cells derived EVs, including membrane/cytoskeletal proteins, lipids, mRNAs, non-coding RNAs, et al., which possess significant roles in regulating bone homeostasis and may serve as biomarkers for bone disease diagnosis.^52^ Therefore, interventions targeting these cargos seem to be a promising therapeutic strategy for bone diseases.

Osteoarthritis, a prevalent, aging-related, and disabling disease, still lacks effective disease-modifying therapies. Recently, Liu et al. identified exosome-mediated subchondral bone-cartilage crosstalk as a potential target for OA therapy.^200^ In their study, a series of microRNAs (miRNAs) was found to be obviously upregulated in bone marrow osteoclasts from OA mouse models after the surgery, including miR-21a-5p, miR-214-3p, miR-148a-3p, miR-199a-3p, miR-378a-3p and several miRNA families such as miR-30 (miR-30a-5p, miR-30c-5p, miR-30d-5p, and miR-30e-5p), miR-200 (miR-200b-3p and miR-200c-3p), and miR-29 (miR-29a-3p and miR-29b-3p). Among them, the four most upregulated miRNAs and miRNA families (miR-21a-5p, miR-214-3p, miR-30a-5p, and miR-30d-5p family) were consistently upregulated in subchondral bone osteoclasts, serum circulating exosomes, and serum osteoclast-derived exosomes in OA mice and in OA patients, compared with sham-operated mice and healthy individuals. Decreasing osteoclast-derived miRNA expression by deleting Dicer (a key miRNA-processing enzyme^201^) or blocking osteoclast-derived exosomes using D-Asp_8_-mediated osteoclast-targeted delivery system containing siRNA of Rab27a (a key intracellular molecule mediating the fusion of multivesicular body to the plasma membrane^202^), substantially attenuated the OA progression in murine models, with an improved matrix degradation, osteochondral angiogenesis, and sensory innervation, in cartilage, by inhibiting tissue inhibitor of metalloproteinase 2/3 (TIMP 2/3). Moreover, the authors screened LJ001 (Fig. 7c) as a unique, low-toxic Rab27a-inhibiting small molecule for osteoclasts with minimal influence on osteoclastogenesis and bone resorption activities and synthesized D-Asp_8_-LJ001 to enhance its osteoclast-targeting ability, which achieved a prominently enhanced therapeutic effect in osteoarthritis, further validating the druggability of osteoclast-derived exosome-mediated crosstalk.

As mentioned in section 2.1, apoptotic bodies derived from osteoclasts containing miR-214-3p can suppress osteogenesis by targeting Osterix and ATF4 and promote osteoclastogenesis through the PI3K/Akt pathway. In another follow-up study by John et al., systemic delivery of recombinant adeno-associated viral serotype 9 (rAAV9) vectors containing anti-miR-214-3p tough decoys effectively reversed estrogen deficiency- and aging-induced osteoporosis, while rAAV9-mediated overexpression of miR-214-3p aggravated bone loss in mouse models.^203^ Notably, miR-214-3p tough decoys administration showed minimal effect on bone remodeling in healthy mice, which indicates the translational potential of miR-214-3p for clinical use in osteoporosis, as being the few bifunctional miRNAs reported regulating both osteoblast and osteoclast activities. In addition, miR-182 is also a worthwhile drug target in bone homeostasis regulation, considering the bone homeostasis improvement by its inhibitors in osteoporosis, rheumatoid arthritis, and physiological conditions through inhibiting the double-stranded RNA-dependent protein kinase (PKR) downstream of RANKL to upregulate interferon-β (IFN-β) expression in macrophages and pOCs, which is a potent autocrine suppressor of early-stage osteoclastogenesis.^204^ Thus, miR-182-targeted therapy may avoid the rebound osteoclast activities of RANKL inhibitors we discussed in section 3.1.1 and realize a more precise regulation of osteoclastogenesis.

Apart from osteoclast-derived exosomes, exosomes derived from other bone cells were also found to participate in bone homeostasis. Very recently, Wang et al. found that BMSC-derived exosomes containing miR-140-3p can promote osteogenesis by inhibiting Plexin-B1 expression and downregulating the Plexin-B1/RhoA/Rock signaling pathway.^205^ It was also proven to promote osteo/dentinogenic differentiation of human dental pulp stem cells by inhibiting lysine methyltransferase 5B (KMT5B),^206^ which also indicated its potential in bone tissue regeneration. In addition, miR-31a-5p derived from aging BMSCs with bone formation inhibition and bone resorption promotion ability^207^ and miR-155 secreted by vascular endothelial cells with osteoclastogenesis inhibitory effect^208^ can be promising targets for bone homeostasis regulation. Nevertheless, drug delivery obstacles, such as off-target effects, degradation by internal nucleases, toxicity, and immunogenicity continue to present challenges to further apply miRNA-targeted therapy, especially for bone diseases. In John’s study, it was surprising to find inapparent side effects on other organs considering the broad participation of miR-214-3p in numerous biological processes, including skeletal development, immune responses, oncology, tumor growth, angiogenesis, and cardiovascular ischemic injury, which may be attributed to the special affinity of rAAV9 vectors for osteoclasts and osteoblasts.^209^ Therefore, an additional modification on the vector of a bone-targeting moiety may further increase their transduction efficiency and safety, which was confirmed in another study from the team.^210^

#### Gene expression

##### Sp7

Considering its critical role in sensing mechanical loading and passing anabolic signals, regulation targeting the osteocyte dendrite network seems promising to maintain bone homeostasis. Apart from undergoing apoptosis and turning into bone-lining cells, late-stage osteoblasts will differentiate into osteocytes. To be deeply embedded into the bone osteoid, proteolytic activity is required. Collagenase and MMPs are required for this process, and the existence of collagenase-resistant type 1 collagen and the deletion of MMPs were reported to inhibit osteocyte network formation.^211,212^ Evidence also showed that a similar order of complexity between the dendrite network and neuron connections in the brain could be observed.^213^ However, despite a deepening knowledge of its function and formation process, little is known about the underlying regulatory mechanism. Recently, Wang et al. reported the role of the transcription factor Sp7 and its target gene Osteocrin in regulating osteocyte dendrites.^214^ Sp7-deleted mice showed higher cortical porosity and decreased bone mineral density in their study owing to reduced osteocyte dendrites and inter-osteocyte connectivity. Increased osteocyte apoptosis and empty lacunae, and subsequently induced high RANKL, were also observed. Dendrite numbers were even reduced in nonapoptotic osteocytes. Osteocrin overexpression, in contrast, reversed these defects. These results indicate that Sp7 may be not only a crucial factor for Osterix-mediated early-stage osteoblast differentiation and related to osteogenesis imperfecta but also a continuing key regulator in maintaining osteocyte dendritic development.^215^

##### Runx2

As a master transcription factor, Runx2 plays a vital role in bone formation.^216^ From mesenchymal stem cells to immature osteoblasts, the expression of Runx2 increases in pOBs but is downregulated in mature osteoblasts. By directly regulating the hedgehog, WNT, FGF, et al., signaling pathways, Runx2 induces the osteogenic differentiation of BMSCs.^216^ During osteogenesis, it can upregulate the expression of genes encoding OCN, ALP, and type 1 collagen.^217^ Genetic defects in Runx2 can cause craniofacial malformations characterized by open fontanel, while gain-of-function of fibroblast growth factor receptors (FGFRs) upstream of Runx2 leads to premature suture obliteration.^218^ Positive and negative regulators via gene expression, protein–protein interactions (PPIs), and posttranslational modification of Runx2 have been emphasized recently. Yang et al. found that exosomes derived from osteoclasts containing miR-23a-5p can inhibit osteogenic differentiation,^219^ while miR-365-3p promoted osteogenic differentiation by targeting Runx2 in osteoporosis.^220^ A comprehensive understanding of the miRNA regulation of Runx2 in osteoblast differentiation would help select more effective targeting sites at the gene level, which was reviewed by Narayanan et al.^221^ In addition, Runx2 can be upregulated through protein–protein interactions by factors such as BMPs, FGFs, and osteoclast-derived apoptotic bodies containing RANK and downregulated by the Snail protein and twist transcription factors.^222,223^ Our previous research also found that Krüppel-like factor 2 (KLF2), a zinc finger structure and DNA-binding transcription factor, could promote osteoblast differentiation by physically interacting with Runx2.^224^ Posttranslational modification of Runx2 is another significant process, and potential targets of Runx2-modifying enzymes through phosphorylation, prolyl isomerization, acetylation and ubiquitination for bone diseases were summarized in Kim’s review.^218^

### Undruggable targets

Over the past 40 years, the survival of osteosarcoma has stagnated due to a common resistance to neoadjuvant MAP (methotrexate, adriamycin, and platinum) chemotherapy, and increasing genomic and functional studies of osteosarcoma have emerged, expecting to exploit new drug targets.^225^ Among them, tumor-suppressor genes such as p53 (TP53),^226^ retinoblastoma (RB),^227^ PTEN^228^ et al., were found to be the significant responsible genes in osteosarcoma genesis and its resistance, with recurrent somatic mutations and copy number alterations. Unfortunately, no therapies targeting these genes have been successfully established in the clinic for osteosarcoma thus far.

Generally, the difficulty in targeting these tumor-suppressor genes derives from the complexity and heterogeneity of their genomic landscape.^229^ For instance, as a transcription factor (TF) associated with cell cycle arrest, apoptosis, and metabolism, wild-type TP53 functions as a potent tumor-suppressor, and its normal function is found lost in 47–90% of osteosarcoma.^226,230^ In addition, among patients with Li–Fraumeni syndrome, a rare autosomal dominant disorder due to TP53 mutations, up to 12% of them develop osteosarcoma.^231^ Mice with TP53 deletion and combined TP53 and Rb1 deletion driven by an osteoblast-based promoter led to a 77 and 100% rate of osteosarcoma.^232,233^ These results indicate a tantalizing gene therapy targeting TP53, noticing its higher disease modulation than signaling proteins. Nevertheless, as a highly disordered TF, there tends to be a huge number of variable gene positions causing missense and nonsense mutations in and beyond the DNA-binding domain, leading to mistaken misfolding and conformation of TP53, which makes it challenging to anchor a universal target, represented by the failure of Eprenetapopt^234^ (APR-246, a small molecule reactivating mutant and inactivated p53 protein) to meet the primary endpoint in a phase 3 trial of TP53-mutant myelodysplastic syndrome. Moreover, the lack of an accessible hydrophobic pocket in the TP53 protein further increases the binding difficulty for small-molecule drugs. Although alternative targeting strategies such as restoration of wild-type TP53 activity by inhibiting upregulated TP53 negative regulators such as E3 ubiquitin ligase murine double minute 2 (MDM2) (e.g., RG7112^235^ and Idasanutlin^236^ (RG7388), two oral MDM2 inhibitors), or inhibiting proteins or signaling pathways that TP53-null or mutated cells highly while wild-type TP53 cells minimally express, were proven to impair sarcomagenesis and cell proliferation.^227,237^ They may not be suitable for most osteosarcoma, considering its relatively lower upregulation of MDM2.^226^ Likewise, although everolimus could increase the therapeutic effect of sorafenib in unresectable relapsed osteosarcoma by inhibiting mTOR signaling downstream of the PTEN/PI3K/Akt signaling pathway, it possessed limited therapeutic effect in osteosarcoma monotherapy.^238^ In addition, side effects such as bone marrow suppression, and gastrointestinal toxicity,^237^ may be attributed to the multiple biological functions of these tumor-suppressor genes, further increasing the difficulty of targeting them in bone sarcoma.

## Delivery targets based on bone structure and remodeling biology

### Delivery targets at the bone tissue level

Commonly, mature skeletons consist of organic matrix (20–40%), water (5–10%), lipids (<3%), and inorganic minerals (50–70%).^239^ The organic part represents approximately 30% of the total dry bone mass, primarily consisting of collagen fibers, glycoproteins, proteoglycans, and other proteins.^240^ Collagen fibers constitute the framework of the extracellular matrix, where cells migrate and produce secretions such as ALP, type 1 collagen, cathepsin K, TRAP, and MMPs. The inorganic content consists of hydroxyapatite [Ca10(PO4)6(OH)2], carbonate, acid phosphate, and magnesium. As the major inorganic component, bone hydroxyapatite (HAP) is featured by its smaller crystals and lower crystalline compared with geologic hydroxyapatite crystals with the largest dimension of approximately 200 Å, which enables easier mineral renewal. During bone remodeling, the crystallinity and surface properties of HAP vary on the resorbing lacuna and bone formation site: the bone-forming surfaces covered by osteoblasts are characterized by low crystalline hydroxyapatite along with amorphous calcium phosphonate while the resorbing lacuna covered by osteoclasts is characterized by highly crystalline hydroxyapatite.^241^

## Bone matrix

### Hydroxyapatite

#### Bisphosphonates and their analogs

As analogs of endogenous pyrophosphate, BPs can chelate with deviant calcium ions (Ca^2+^) present in HAP, forming strong bidentate or tridentate bonds through their P-C-P structure.^242^ The other two groups on the P-C-P carbon atom, R1 and R2 (by which bisphosphonates are classified), have been well established to further influence the affinity and the anti-resorption ability. According to the studies by Nancollas et al., with the same P-C-P bond and OH in R1, the affinity for HAP still differs with a rank order of highest to lowest for the bisphosphonates studied of zoledronate > alendronate > ibandronate=risedronate> etidronate (Fig. 7b).^243^ Russell et al. further demonstrated that the nitrogen side groups could directly bind to the hydroxyl groups on the HAP surface.^244^ Both the angle and distance of the N-H-O bond can alter their binding, and the optimal affinity is reached with a bond angle of approximately 125° and a bond distance of 3 Å, which can explain the higher affinity of alendronate (132°, 2.7 Å). In addition, the binding affinity is also influenced by changes in the zeta potential of the HAP surface after the adsorption of BPs. The positively charged nitrogen-containing R2 can turn the charge on the surface of HAP into a more positive potential, thus attracting more negatively charged phosphonate groups and enhancing the binding capacity. Additionally, an alteration of the R2 group can result in different antiresorptive effects, depending on whether the side chain contains nitrogen and its structure. BPs with a nitrogen heterocyclic ring of R2 (such as risedronate and zoledronate) tend to be the most potent, while BPs with a basic primary nitrogen atom in an alkyl chain (e.g., alendronate and pamidronate) are inferior to BPs with more highly substituted nitrogen, such as ibandronate, whereas they all surpass BPs with no nitrogen in R2 (e.g., clodronate and etidronate).

When applied as targeting ligands for drugs or vectors, BPs, and vectors can be conjugated with or without linkers.^245^ Their conjugation should not modify the properties of BPs or drugs. Commonly, the R1 or R2 group is preferred for conjugating vectors to retain Ca^2+^ chelating ability.^246^ Although choosing BPs with higher HAP affinity yields a stronger targeting ability, BPs with lower binding affinity may be more advantageous in drug dissociation and multiple bone loci acting. Similarly, a higher mol% of BP in the vector can lead to stronger binding but higher negative zeta potential, thus reducing circulating time and increasing liver distribution. In contrast, a lower mol% may generate a balance between pharmacokinetics and bone binding, thus actually achieving a higher bone/liver distribution. In Vanderburgh et al.’s research, 10 mol% alendronates exhibited the highest accumulation ratio at the bone tumor site.^247^ In addition, BPs can serve as the primary framework of the vector. Recently, a rational design of BP lipid-like materials for mRNA delivery to bone was developed.^248^ Through ligand substitution, BP-lipid, DOPE, cholesterol, and C14PEG2000 constituted the BP-LNP (lipid nanoparticles) with different mRNAs encapsulated. BP-LNPs exhibited much lower biodistribution in the liver and spleen and higher bone marrow/surface accumulation and cellular uptake than LNPs without BP. Intriguingly, histological staining showed that enhanced green fluorescent protein (EGFP) transfection signaling was mainly detected in the bone marrow, especially in the endosteum, rather than on the bone surface. BP-LNPs encapsulating BMP-2 mRNA showed a prominent increase in BMP-2 expression on the bone surface and bone marrow compared with LNPs without BP. Although lacking verification in a disease model, these results indicate that BP-modified nanoparticles can be a promising method for bone-targeted drug delivery. Moreover, the BP ligand can also exhibit an additional anti-resorption effect in the delivery system by inhibiting osteoclasts or farnesyl diphosphate synthases.

Owing to these properties, BP ligands have been broadly exploited in targeted drug delivery in preclinical studies for multiple bone diseases.^245^ In targeted antimicrobial therapy for bone infection, BP-antibiotic conjugation prominently decreased minimum inhibitory concentration and prolonged the duration of the therapeutic effect.^249^ In bone tumor-targeted drug delivery, BP conjugation can greatly increase the concentration of antitumor drugs in bone and reverse bone mechanical properties by inhibiting bone resorption.^250^ Nevertheless, despite the higher accumulation in bone and lessened side effects on other tissues, it still cannot avoid the toxicity of antineoplastic drugs to normal bone cells for lack of tumor cell specificity. In view of this, an improvement strategy was recently proposed by TIAN et al.^251^ By conjugating alendronate with HER2-targeted antibody Tras using pClick technology, bone metastasis-targeted Tras-Alen was synthesized. Administration of it in mouse models showed a significantly higher accumulation of Tras-Alen in bone than in other organs, but a prominently decreased distribution in healthy bone tissue than in cancer-bearing bones, which not only confirmed the role of bone-targeted ligands for bone diseases but also indicated the feasibility for drugs to further target specific site after reaching bone by dual-targeting or multiple-targeting. Of note, the anti-resorption effect of BP ligands seems controllable. In Guan et al.’s study, a one-tenth therapeutic dose of alendronate in the delivery compound exhibited no observed antiresorptive effect during their study period.^252^ However, further investigations are required, considering the long half-life of BPs in bone (more than 10 years).^253^

Apart from BPs, compounds with similar P-C-P structures have also been exploited as therapeutic and delivery ligands. Phytic acid (PA), also known as inositol hexakisphosphate, which naturally exists in cereals, fruits, vegetables, and mammalian cells,^254^ was reported to possess inherent anticancer,^255^ anti-osteoclastogenesis,^256^ and HAP-targeting abilities (Fig. 7a).^257^ In Wang et al.’s study, the hydrolysis product of cisplatin was used to react with the phosphate group on PA, constructing cisplatin-PA nanoparticles whose surface was covered by residual phytic acid phosphate groups.^258^ In vitro experiments on MDA-MB-231 cells and in vivo experiments on a bone metastatic breast cancer model both showed therapeutic efficacy and inhibited the osteoclast differentiation of bone marrow monocytes induced by tumor-secreted RANKL and M-CSF. Surprisingly, the phosphate-platinum linkage showed a pH-responsive characteristic with a significantly promoted release of cisplatin at pH 5.0 compared with pH 7.4, which can not only suit the acidic microenvironment of bone tumors but also alleviate the toxicity of cisplatin probably through a lower administration dose. Compared with delivery systems based on liposomes and polymer nanoparticles, PA nanoparticles may raise fewer safety concerns due to their endogenous presence and easy clearance from the human body due to their small size. Apart from being synthesized as nanoparticles, PAs can also serve as targeting ligands modified on other vectors. In the research by Zhou et al., PA-capped platinum nanoparticles were synthesized. Both in vivo and in vitro analyses showed efficient bone tumor growth inhibition and alleviation of tumor-associated osteolysis after administration.^257^ In view of the similar targeting mechanism and regulation of bone remodeling, a comparison between them is attractive. Contrary to bisphosphonates, especially nitrogen-containing ones, phytic acid showed less interference with upper gastrointestinal disturbance.^259^ With regard to the HAP dissolution inhibition ability, phytate acid was similar to alendronate and greater than etidronate.^259^ Unfortunately, no published studies to date have analyzed the distinction between them as delivery ligands.

#### Tetracycline

Tetracycline (TC) is a well-known broad-spectrum antibiotic with an ABCD naphthacene ring basic skeleton (Fig. 7a) and has been used to treat bacterial infections, periodontitis, and dermatosis for decades. Notably, TC exhibits a high affinity for the bone mineral matrix, especially in bone with a high remodeling rate, making it a valuable tool for bone imaging and quantifying new bone formation by labeling the surface of growing bone due to its fluorescent properties.^260,261^ Therefore, choosing TC as a bone-targeted ligand can be appropriate for bone diseases with a high bone turnover rate, e.g., osteoporosis.^262^ Mechanistically, TC binds Ca^2+^ in hydroxyapatite by the hydroxyapatite binding domain, which is formed by the phenolic β-diketone group attached to carbons 10 and 11, the enol group at carbons 1 and 3, and the carboxamide group attached to the acylamino at carbon 2.^263^ van der Waals attractions and hydrogen bonding between the hydroxyl groups of HAP and TC also contribute to the surface complexation between TC and HAP.^264^ Current research mainly focuses on simplifying the structure of TC to reduce potential side effects due to its biological activity. The emphasis of structure simplification focuses on retaining the ticarbonylmethane group in the A ring of TC, which is the core binding part.^265^ The feasibility of simplification was also proven by the fact that 3-amino-2,6-dihydroxybenzamide retained 50% affinity for TC.^266^

Utilizing the positioning effect of TC on the bone surface, Lin et al. synthesized smart nanoparticles composed of a sodium bicarbonate-containing layer and TC-functionalized nanoliposomes (NaHCO3-TNLs) as bone-targeted antacids.^267^ Administration of NaHCO3-TNLs suppressed the initial acidification of osteoclasts in vivo and generated a chemically regulated biocascade to bone remodeling by promoting osteoclast apoptosis, in which the apoptosis-derived extracellular vesicles containing RANK further consumed serum RANKL and coupled bone formation.^53^ In addition, TC-grafted methoxy polyethylene glycol (mPEG)-poly(lactic-co-glycolic acid) (PLGA) micelles carrying astragaloside IV (AS, the main active component of astragalus membranaceus, a natural antioxidant that suppresses osteoclastogenesis by inhibiting the ERK pathway) were synthesized.^268^ After administration in mice, much higher fluorescent intensities in the femurs of mice and improved pharmacokinetics data of the TC-mPEG-PLGA group were observed compared to the mPEG-PLGA control group. However, liver distribution was still the highest among the tissues, suggesting that depletion from the mononuclear phagocyte system (MPS) is still a major consumption for the drug delivery system. Ackun-Farmmer et al. further illustrated the depletion owing to MPS in bone-targeted drug delivery: a higher administration dose can lead to a higher accumulation in bone tissue; however, the ratio in other MPS tissues would be greater.^269^ In contrast, macrophage depletion mediated by clodronate liposomes decreased nanoparticle accumulation in the liver and lung while improving its concentration in bone. To improve evasion from the MPS system, achievements have been made in designing PEGylation and zwitterionic surface chemistries of the vectors.^270^ In addition, attempts were also made to modify vectors with mimic peptides of CD47, a ‘marker of self’ membrane protein that binds to CD172α (S1RPα) on phagocytes to reduce depletion from MPS during HSC homing and other migration processes (Fig. 2b).^271^ Notably, CSF1R inhibition can lead to macrophage depletion in other tissues and further block the replenishment of macrophages.^272^ Considering that the obstruction of the monocyte system is the main obstacle for targeted drug delivery, the combination of CSF1R inhibitors in the drug delivery system may possess dual functions of anti-osteoclastogenesis and reducing liver- and lung-induced drug depletion; however, to the best of the author’s knowledge, no such attempt has been reported.

Despite the skeletal affinity, the impacts of TC on bone remodeling remain enigmatic.^262^ TC has been shown to possess an anti-collagenolytic ability that inhibits collagenase and alleviates bone resorption. In addition, TC also upregulates the expression of procollagen mRNA, thus activating more osteoblasts.^273^ Nevertheless, TC seems to have a dose-dependent effect on osteoblastogenesis, with a low dose (1 μg/ml) of doxycycline or minocycline promoting the proliferation of osteoblastic cells without affecting their functional activity, while higher doses (≥5 μg/ml) suppress osteoblast function.^274^ Given the permanent chelation of TC to calcium, its cellular impacts need to be further investigated for usage as a target ligand. In addition to indeterminate cellular influence, potential side effects such as tooth staining and enamel hypoplasia raise safety concerns for its usage in pediatric-associated bone diseases. Moreover, tetracycline possesses low chemical stability, especially when conjugating with drugs or nanocarriers. These drawbacks may render TCs less optimal as targeting ligands in bone-targeted drug delivery.

#### Hydroxyapatite-targeted peptides

##### Acidic oligopeptides

Inspired by the specific affinity of noncollagenous proteins in the bone matrix, such as OPN, OCN, and bone sialoprotein, to the resorption surface, acidic oligopeptides (AOs) consisting of aspartic acid (Asp) or glutamic acid (Glu) have been identified and widely applied as targeting ligands for drug delivery to bone (Fig. 7a).^275–278^ Although the binding mechanism is still under debate,^279^ the negative charge (the COOH group) and polarity of the side groups in these amino acids (AAs) appear to be responsible for their ability to bind to calcium ions.^280^ Notably, the affinity between acid oligopeptides and HAP was not altered by the species (Asp or Glu) or their optical antipodes (L or D), only by the type of polymeric amide bond and the number of AA residues.^281,282^ Poly-(a-aspartic/glutamic acid) shows higher chelating ability than poly-(β-aspartic/glutamic acid) and poly-(a, β-aspartic/glutamic acid). An increase in the polymer chain results in enhanced affinity and the dissociation constant (*K*_d_), suggesting that polymers containing longer chains are better for binding. However, for vector conjugation, the increase in chain length does not equal linear affinity growth. The size of the vector also plays a vital role in the absorption process, and the most commonly recommended number of repeated AAs was generally 6-8.^281^ In addition, its spatial configuration matters for vectors. According to research by Nielsen et al.,^283^ linear peptides were 2.7 times more concentrated in bone than branched peptides. Meanwhile, the side chain of AAs also counts. Since molecular orbital studies chelate calcium optimally when the proximal anionic charges separate by a distance of 8.6 Å,^284^ Asp with a single carboxylic acid side and Glu with two carboxylic acid side chains are the best choices. Despite their equal affinity, Glu may be preferred owing to the aspartamide impurities spontaneously formed by poly-asp,^285^ thus resulting in reduced purity.

In the research by Liu et al., a D-Asp_8_-conjugated liposome carrying antagomir-148a (miR-148a, a gene that promotes osteoclastogenesis) was developed, and its administration attenuated bone resorption and improved the deteriorated trabecular microstructure in OVX mice.^286^ The successful bone resorption surface targeting ability was confirmed by the much higher colocalization of FAM-labeled antagomir-148a and osteoclast-associated receptor positive (OSCAR^+^) pOCs and mOCs compared to other groups by immunofluorescence analysis. In addition, labeling of rhodamine B-conjugated D-Asp_8_ was found at the eroded surface rather than the calcein green-labeled bone formation surface, and TRAP staining further verified that rhodamine B-labeled bone surfaces were occupied by osteoclasts. Similarly, polyurethane (PU) nanomicelles modified by Asp_8_ containing anti-miR-214 were developed later.^287^ As mentioned above, miR-214 is a potent inhibitor of osteogenesis and an activator of osteoclastogenesis secreted by later-stage osteoclasts. Administration of the PU-Asp_8_ system prominently improved bone microarchitecture and bone mass in OVX mice.

In fact, although AA-mediated delivery targets the bone resorption surface occupied by OSCAR^+^ cells, therapeutic agents targeting other bone cells can also benefit from this system. In the research by Huang et al., icaritin (Fig. 7c), a traditional Chinese medicine extract that inhibits the adipogenic differentiation of BMSCs and promotes osteogenesis through the Akt/GSK3β/β-catenin signaling pathway, was encapsulated in a bone-targeted liposome containing an oligopeptide of eight aspartate residues (Asp_8_).^288^ Enhanced osteoid and new bone formation and decreased adipocyte area of the fifth vertebra of the lumbar were detected compared to the icaritin-liposome control group in OVX mice. However, despite the confirmed delivery efficiency of the Asp_8_^+^ liposome group, evidence also showed that Asp-conjugated cholesterol-containing liposomes (~6%) could increase serum cholesterol levels and thrombus areas in the bone marrow, which might dysregulate lipid metabolism and cause adipose accumulation.^289^

In addition to these preclinical drug delivery trials, acidic oligopeptides have also been applied in the clinic. As a rare, genetic, and progressive metabolism disorder characterized by impaired mineralization due to inborn low serum ALP activity, hypophosphatasia is a hard-to-treat disease. Distinguished from osteoporosis in low bone mass, hypophosphatasia is caused by deficient bone mineralization rather than excessive bone resorption. Therefore, it is less likely to benefit from anti-resorption agents such as bisphosphonates and denosumab. Bisphosphonates are even theoretically contraindicated, considering their further inhibition of TNSALP activity due to their inorganic pyrophosphate-like structure.^290^ The therapeutic effect of bone anabolic medications such as romosozumab and teriparatide in adult hypophosphatasia was uncertain in clinical trials. Some participants were reported to show a short-term duration of BMP and ALP increase (usually <2 years),^290,291^ while some other participants had not,^290,292^ indicating that the anabolic response may be associated with specific gene mutations.^293^ Such imprecise therapeutic conditions were not improved until the emergence of asfotase alfa, a bone-targeted recombinant tissue-nonspecific alkaline phosphatase (TNSALP) composed of the catalytic domain of human TNSALP, an IgG1 Fc fragment, and a deca-aspartate motif for hydroxyapatite binding.^294^ Since 2015, subcutaneous asfotase alfa (Strensiq^TM^) has been approved by the FDA for long-term therapy of pediatric-onset hypophosphatasia.^295^ Multiple noncomparative clinical trials have validated its remarkable therapeutic effect with sustained improvements in bone mineralization (> 3 years), muscle strength, cognitive development, et al.^296^ Moreover, it is well-tolerated and has mild to moderate treatment-related adverse responses.^296^

##### DSS peptides

Through dentin extracellular matrix mineralization experiments, small peptides with repeats of the tripeptide aspartate-serine-serine (AspSerSer, also known as DSS) were identified to specifically bind to hydroxyapatite.^241^ Similar to AOs, a higher affinity would be attained when the number of DSS repeats increases with an optimal repeat of 6.^241^ However, the DSS peptide targets low-crystallized hydroxyapatite where bone formation occurs, which may result from phosphorylation during combination. Hence, when DSS is used as a targeting ligand in drug delivery, osteogenic-lineage cells on the bone formation surface will be selectively affected. In 2012, Zhang et al. first attached (AspSerSer)_6_ to cationic liposomes containing pleckstrin homology domain-containing family O member 1 (Plekho1, also known as casein kinase 2 interacting protein (CKIP-1), an intracellular promotor of bone formation, muscle cell differentiation, and tumor cell proliferation) siRNAs targeting bone formation surfaces and improved bone formation in an osteoporotic rat model.^297^ In their research, a comparison between FITC-labeled (AspSerSer)_6_ and FITC-labeled (Asp_8_) injection after preinjection of xylenol orange (a red fluorescent calcium-binding dye capable of labeling new bone deposition at bone-formation surfaces) in rats was conducted: the bone formation surface was largely labeled with (AspSerSer)_6_ whereas little Asp_8_ was observed. Likewise, the bone resorption surface was labeled with Asp_8_ while very little (AspSerSer)_6_ was observed. In addition, their coinjection showed little colocalization. The in vivo targeting effect was analyzed using biophotonic imaging technology: the intraosseous fluorescence signal of FAM-labeled Plekho1 siRNA was strongest in the (AspSerSer)_6_-liposome group, and it decreased the hepatic fluorescence signal, which was intense in the jetPEI (a commercialized in vivo transfection reagent for nucleic acids) and liposome alone groups. Plekho1 protein and mRNA expression in bone and nonskeletal tissue (e.g., the liver, kidney, and lung) were also significantly lower and higher, respectively.

##### Comparison between HAP-targeted ligands

**Fig. 6:**
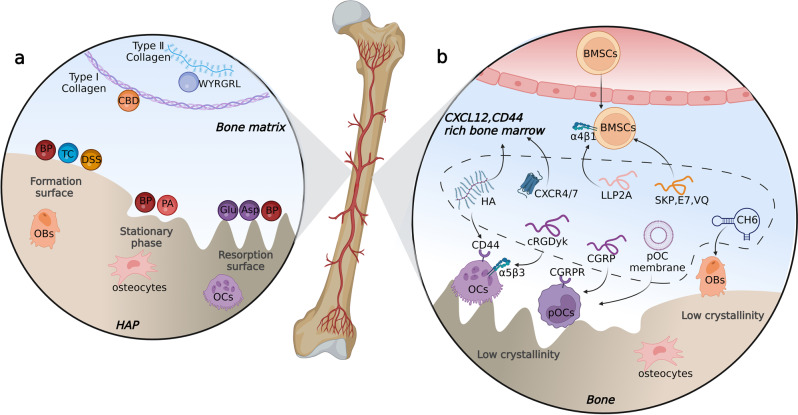
Bone-targeted drug delivery ligands. **a** Bone matrix-targeted ligands. TC and DSS target the bone formation site, while Asp and Glu target the resorption site. BPs and PA are less influenced by surface property. CBD and WYRGRL target type I and II collagen, respectively. **b** Bone marrow- and bone cell-targeted ligands

As the most frequently used HAP-targeted ligands, BPs, TC, AOs, and DSS have been featured in many preclinical studies in recent years as promising auxiliary sections for more precise bone-targeted drug delivery. (Table 1) Nevertheless, distinctions among them may induce precedence of each ligand for a different therapeutic condition. First and foremost, the HAP site they bind to varies: AOs prefer higher crystalline HAP, TC, and DSS prefer low crystalline HAP, and BPs are less influenced by the crystallinity.^298,299^ As mentioned above, the physicochemical characteristics of bone-formation surfaces covered by osteoblasts are low crystalline hydroxyapatite and amorphous calcium phosphonate, while the bone-resorbing surface covered by osteoclasts is highly crystalline hydroxyapatite.^241^ Hence, when conjugating ligands above for bone-targeted drug delivery, the pharmacological sites of the drugs may also count (Fig. 6).

**Table 1:** Delivery ligands targeting bone matrix

Ligand	Binding feature	Advantage	Disadvantage	Ref
Bisphosphonate	Less influenced by the crystallinity of HAP	Higher binding ratio Intrinsic anti-resorption effect Easy conjugation with drugs or drug vectors through R1 or R2 Economic convenience	Long-term presence in HAP Side effects such as ONJ, atypical femoral fractures, esophageal cancer, and nephrotoxicity Potency to reduce the secretion of insulin-like growth factors and BMPs to promote osteoblast formation	^243,244^
Phytic acid	Less influenced by the crystallinity of HAP	Natural existing compounds in vivo Inherent antitumor and anti-osteoclastogenesis ability Reduced gastrointestinal disturbance	Low oral bioavailability	^258^
Tetracycline	Prefers low crystallinity HAP and growing surface	Intrinsic anti-collagenolytic ability Upregulating the expression of procollagen mRNA, thus activating more osteoblasts Strong affinity toward bone with a high remodeling rate	Teeth staining and enamel hypoplasia Dose-dependent dual effect on osteogenesis Permanent chelation Low chemical stability to conjugate with drugs or vectors	^264^
Acidic Oligopeptide	HAP on bone-resorbing surface	Faster binding rate Better drug release and less side effect due to high biodegradation Usage in virus vectors	Not orally bioavailable Higher drug release but lower concentration due to lysis of enzymes to linkages	^275,286,288^
DSS	HAP on bone formation surface			^241,297,346^
Collagen-binding domain (CBD)	Collagen in bone	Easy combination for agents, cell adhesion and retention effect, especially when used in implant coating	Distribution in skin	^306^
WYRGRL	Collagen II	Cartilage specific	Much lower collagen II content in cartilage than skin Rely on intra-articular injection	^312^

Regarding delivery characterizations, as biologically degradable peptides, AOs and DSS would not form colloids with calcium ions, which enables more efficient drug release in bone and easier excretion by kidneys, thus possessing fewer unexpected long-term side effects but shorter circulating time compared to BPs and TCs. In addition, distinctions have been observed in their binding rate and strength. Murphy et al. demonstrated that the binding rate of AO is faster than that of BP (there was a statistical difference in bone distribution from 0.5 to 1 h after administration),^300^ which could be a result of the negative net charge of AO and the smaller contact area of BP. Meanwhile, BPs had a higher binding ratio, which may be attributed to their specific binding.^298^ Moreover, TCs were reported to be inferior to AOs in bone fracture targeting.^283^ From the author’s perspective, binding strength may play a more significant role in targeted therapy for skeletal diseases such as osteoporosis and bone tumors, while a faster binding rate may be helpful in conditions such as arthritis and bone infections, where there may exist an acute inflammatory phase. In addition, oral administration may not be suitable for AO-mediated drug delivery due to easy gastrointestinal degradation. Intravenous administration may be more effective; however, it may discourage the compliance of patients.

Of note, the vector type in the delivery system is also a significant factor to consider for selecting hydroxyapatite-targeted ligands. Although these ligands have been proven feasible in non-virus vectors such as liposomes, polymer particles, micelles, and dendrimers for bone diseases,^301^ it may be a different condition in viral vectors-based delivery for that bisphosphonates and TC are synthetic organic compounds without encoding genes. In the study by Yang et al., injection of rAAV9 vectors grafted with bone-targeting peptide motif (AspSerSer)_6_, carrying an artificial-miRNA that targets Shn3 (a gene that downregulates bone formation by promoting Runx2 degradation, suppressing the WNT signaling pathway, and inhibiting the type H vessel-coupling SLIT3) substantially enhanced osteogenic differentiation and improved trabecular structure in OVX mice with decreased distribution in other tissues.^210^ In particular, the DSS ((AspSerSer)6) peptide motif was grafted onto the N-terminus of the VP2 subunit of the AAV9 capsid protein, generating markedly higher genome copies in the hydroxyapatite pellet and a retained transduction efficiency, which is a remarkable job for not only first identifying serotype 9 as the bone-tropic type among AAV vectors (mainly transduce osteoblasts, osteoclasts, and osteocytes) but also revealing the feasibility and efficacy of inserting bone-targeted-peptides-encoding DNA sequences in rAAV9 to further enhance its osteotropism, considering the off-target risk of rAAV9 by traversing the blood-brain barrier and transducing myocardium and striated muscle, liver, and retina.^210,302^ Such systems were further proven to possess exciting preclinical therapeutic effects in murine models of osteoporosis and heterotopic ossification,^203,303^ which indicated the foreseen ponderance of hydroxyapatite-targeted peptides and bone-tropic rAAV9-mediated gene therapy in bone diseases.

##### Collagen

As the major component of the organic matrix, collagen plays a vital role in bone remodeling and osteoporosis.^304^ Collagen-binding domains (CBDs), which are found in the collagenolytic proteases of microorganisms, have been proven to possess a particular affinity for collagen.^305^ Via standard molecular biology techniques, the cDNAs of CBDs can be fused into the N-terminus or C-terminus of applicable proteins and structure-retained proteins with collagen-binding ability can be synthesized by expressing the recombinant protein. In the research conducted by Ponnapakkam et al.,^306^ a fusion protein of PTH (1–33) and a CBD derived from Clostridium histolyticum of Col H collagenase (PTH-CBD) were synthesized to target collagen-rich bone matrix. Monthly administration of PTH-CBD showed a longer duration of spinal BMD improvement, increased ALP levels, and less hypercalcemia or osteosarcoma risk in mice compared to weekly administration of PTH (1–34). Additionally, it vastly decreased the kidney distribution of PTH. In a further study,^307^ a single injection of PTH-CBD achieved a persistent improvement in BMD for up to 12 months, which confirmed its efficiency in sustaining release. However, as the main structural protein component of the extracellular matrix, collagen is also abundant in other tissues, such as skin, tendons, ligaments, cartilage, and blood vessels.^308^ The biodistribution assay validated its nonnegligible concentration in the skin after administration.^307^ In another study of the effect of PTH-CBD in an alopecic mouse model induced by chemotherapy, PTH-CBD promoted hair growth and led to an apparent increase in the number of anagen VI follicles,^309^ which may be attributed to the positive effect of PTH-CBD on WNT signaling in the skin with increased production of β-catenin, an activator of the hair cycle.^310^ In addition, the effect of PTH-CBD on BMD improvement still has an anabolic limit of approximately 2 years, which may restrict its long-term usage.^306^ Notably, apart from drug delivery, CBD modification on implants can improve superficial properties due to its biocompatibility, easy combination with agents, and cell adhesion and retention effect: implants modified with CBD containing the core functional amino acid sequences of laminin α4 were synthesized with enhanced MSC adhesion, angiogenesis, and bone formation effects.^311^ Apart from CBD, collagen II-specific peptide WYRGRL was reported to enhance the cartilage-targeting property of drug vectors,^312^ which may contribute to the development of novel targeted drugs in osteoarthritis therapy.

#### Bone marrow

Stem cell homing refers to the ability of circulating or implanted stem cells to return to the bone marrow niche. Both mesenchymal and hematopoietic stem cells (HSCs) can conduct this process. Successful MSC homing can benefit bone formation, and emerging studies have revealed the therapeutic effect of MSC homing in skeleton-related diseases: BMSCs from osteoporotic patients or from aged and OVX mice revealed reduced migration and invasion ability, while administration of allogeneic or autologous bone marrow-derived MSCs improved bone formation in mice subjected to tibia transverse osteotomy.^313,314^ Nevertheless, the homing ability of endogenous or transplanted MSCs to the bone marrow niche is generally faint.^315^ To enhance homing, efforts have been made to target the specific molecular interactions mediating the process.

Generally, MSC homing is a complicated process taking five steps: tethering and rolling, activation, arrest, transmigration or diapedesis, and migration.^316^ Selectins expressed on endothelial cells facilitate the tethering step. MSCs express CD44 to catch on the selectins and initiate the rolling step without expressing the hematopoietic cell E- and L-selectin ligand (HCELL) or P-selectin glycoprotein ligand-1 (PSGL-1), which are potent ligands in HSPC osteotropism.^317^ Although the exact selectin for MSCs remains unclear, progress has been made to convert CD44 on MSCs into HCELL via a-1,3-fucosyltransferase sugar modification^317^ or fucosyltransferase VI (FTVI) transfection^318^ to enhance their osteotropism. The activation step is facilitated by G protein-coupled chemokine receptors such as CXCR4 and CXCR7, especially in response to inflammatory signals.^319^ As the only ligand of CXCR4 and CXCR7, CXCL12 is a vital signaling protein expressed by marrow stromal cells and endothelial cells.^320^ CXCR4/CXCR7 and CXCL12 constitute the CXCL12/CXCR axis, which is one of the key regulatory signals in MSC homing and reinforcing bone repair in vivo.^321^ Following activation, integrins facilitate the arrest step. Integrin α4β1 (VLA-4) is expressed by MSCs, which respond to chemokines such as CXCL12, and can bind to vascular cell adhesion molecule-1 (VCAM-1) on endothelial cells.^178^ MSCs also express ICAM-1, an integrin ligand, to improve the process (Fig. 2b). Antibodies against β1-integrin inhibit MSC homing and overexpression of a4-integrin, a component of VLA-4, enhances the process.^322^ In addition, LLP2A, an α4β1 specific peptidomimetic, was synthesized, and its delivery by alendronate (LLP2A-Ale) prevented bone loss in both xenotransplantation and immunocompetent mice,^252^ suggesting that it could be a robust homing ligand for transplanted and endogenous stem cells.

In a study by Chen et al., alendronate-modified liposomal nanoparticles carrying the SDF-1 (CXCL12) gene (Aln-Lipo-SDF-1) were developed, and lateral tail vein injection improved bone regeneration in osteoporotic mice.^323^ Alendronate conjugation increased the accumulation of nanoparticles in bone tissue, and encapsulated SDF-1 increased the quantity of green fluorescent protein (GFP)+ MSCs homing to the femoral bone marrow. Despite its role in mediating MSC homing, the effect of CXCL12 on the skeleton is complex and still under debate. Previous studies have indicated that CXCL12 enhances osteoclastogenesis by affecting osteoprogenitor cells and increasing bone resorption in several pathological conditions and that antagonists of CXCR4 improve ovariectomy-induced osteoporosis and multiple myeloma-mediated osteoclastogenesis.^324–327^ However, according to research by Ponte et al., the indirect restraint of CXCL12 on bone remodeling through inhibiting osteogenesis and the osteoclastogenesis support provided by cells of the osteoblast lineage exceeds its direct pro-osteoclastogenic effect.^328^ Additionally, Pont et al. further exemplified that CXCL12 deletion greatly attenuated the loss of cortical bone caused by estrogen deficiency, suggesting that CXCL12 may contribute to estrogen deficiency-induced bone loss. These results raise concerns about the usage of CXCL12-related targeted therapy in postmenopausal osteoporosis.^328^

To realize similar homing effects for drug delivery, modification of these significant intermediates on drug vectors has been attempted. Zhang et al. encapsulated polylactic-co-glycolic acid (PLGA) nanoparticles with the secretome from MSCs to form MSC-Sec NPs and further cloaked them with membranes from CXCR4^+^ human microvascular endothelial cells (HMECs) to obtain CXCR4^+^ MSC-Sec NPs.^329^ Their administration showed a better effect in inhibiting osteoclast differentiation, promoting osteogenic proliferation, and reducing bone loss in OVX rats than CXCR4^-^ MSC-Sec. In addition, CXCR4^+^ MSC-Sec NPs showed sustained and long-term release of OPG and BMP-2 just like real stem cells, which may also validate the role of the secretome in cell-free therapy in regenerative medicine: avoiding the loss in the expression of homing molecules on MSCs after expansion in vitro and safety problems probably related to the transplantation of MSCs such as emboli formation, tumorigenicity, and injections.^330,331^ In another study conducted by Hu et al., exosomes from engineered NIG-3T3 cells that highly express CXCR4 were fused with liposomes carrying antagomir-188 (miR-188, an age-increasing expression gene that promotes adipogenesis and inhibits osteogenesis of BMSCs), forming hybrid NPs (hybrid NPs through fusion with liposomes can enhance loading capacity of exosomes).^20^ The targeting capacity of the hybrid NP was gained through homing recruitment of the CXCR_4_^+^ NP to bone marrow. IV injection of it reversed trabecular bone loss, inhibited adipogenesis, and promoted osteogenesis of BMSCs in an age-related osteoporosis mouse model. Although the liver had the second highest concentration of aggregation independent of the grouping, 48-h and 8-week cytotoxicity analyses showed no abnormalities in the liver along with the heart, spleen, lung, and kidney.

In addition to CXCL12 and CXCR4 modification, a self-assembling peptide SKPPGTSS was identified through phage display as a bone marrow-targeted ligand.^332^ The peptide has a partial (5/7) amino acid sequence homology with a region of CD84, which is expressed on hematopoietic cells and promotes the homing process. Conjugation of SKPPGTSS to a nanofiber hydrogel carrying agomir-29b-5p (an aging-related miRNA that suppresses the expression of matrix metalloproteinases and senescence-associated genes (P16INK4a/P21) via ten-eleven-translocation enzyme 1 (TET1)) promoted cartilage regeneration by suppressing senescence in an osteoarthritis rat model.^333^ Injection of it showed 14-day retention of the encapsulated agomirs in the joint and a much higher fluorescence signal in the joint compared with the control group, which confirmed the effect of the homing-promoting peptide. Surprisingly, the peptide also improved endogenous synovial stem cell recruitment, which achieved the effect of killing two birds with one stone.

### Delivery targets at the bone cell level

Recently, small biomimetic molecules such as peptides and aptamers have been selected for aptamer-drug conjugates (ApDCs) and peptide-drug conjugates (PDCs) to target specific cellular components of bone cells. Compared with bone tissue-targeted ligands, these biomimetic molecules improved the targeting accuracy and decreased the influence on non-target cells (Table 2). In contrast, bone tissue-targeted ligands led by HAP seekers provide potent and stable delivery ability toward bone minerals. Although few studies have compared their merits and demerits in the same delivery system, for a new generation of precision medicine based on small molecules or oligonucleotides, both targeting strategies are promising and can improve their therapeutic effect (Fig. 6).

**Table 2:** Delivery ligands targeting bone cells or bone marrow

Ligand	Targeting feature	Ref
Calcitonin gene-related peptide (CGRP)	CGRP receptor on pOCs	^343^
Hyaluronic acid (HA)	CD44 on the surface of the osteoclasts and the CD44-rich bone marrow microenvironment	^343^
cRGDyk	α5β3-integrin on osteoclasts membrane	^334^
Aptamer CH6	Osteoblasts	^19^
SDSSD	Osteoblast-specific factor 2 (OSF-2)	^346^
LLP2A	Binding integrin α4β1 to improve homing	^252^
CXCR4 or CXCR7	Binding CXCL12 to promote homing to bone marrow	^20,329^
SKPPGTSS (SKP)	Possessing a partial amino acid sequence homology with a region of CD84 to promote homing	^332,333^
VTAMEPGQ (VQ)	Rat mesenchymal stem cells specific	^351,352^
EPLQLKM (E7)	Efficiently interact specifically with MSCs without any species specificity	^349,350^

#### Osteoclasts

Due to the significance of α5β3 in tumor invasion and metastasis, peptide cyclic arginine-glycine-aspartic acid-tyrosine-lysine peptide (cRGDyk) with α5 integrin affinity was synthesized as a tracer for tumor targeting and angiogenesis imaging.^334^ In addition, it could be used as a targeting ligand for vectors in drug delivery, especially in bone metastases. In a parathyroid hormone-induced osteolysis imaging study, an imaging agent targeting osteoclasts was synthesized through conjugation of 64Cu with cRGDyk.^335^ Administration of the PTH complex showed increased specific uptake of osteoclasts on the bone surface and nonspecific uptake of bone marrow macrophages. In another study by Wang et al., a liposomal drug delivery system conjugated with cRGDyk was synthesized to enhance the therapeutic effect of cisplatin in a mouse model of bone metastasis from prostate cancer. Compared with free cisplatin and cGRDyk-free liposomes that function through the enhanced permeability and retention effect (EPR effect, which refers to the phenomenon that molecules or particles of certain dimensions tend to accumulate more in solid tumors than in normal tissues due to high vascularization and large endothelial gaps^336^), cRGDyk-liposomes showed better bone tumor penetration and lower cytotoxicity in vitro and in vivo.^337^

#### Osteoclast precursors (pOCs)

Compared with bone-resorbing mOCs, pOCs secrete PDGF-BB to promote bone formation and angiogenesis of type H vessels through PI3k-Akt-dependent activation of focal adhesion kinase (FAK).^14^ Inhibiting osteoclast activities without affecting pOCs is a challenge for anti-resorption therapy. To date, pH-responsive delivery for bone diseases mainly targets the acidic microenvironment in bone tumors or bacterial films.^338,339^ Surprisingly, by targeting the discriminative extracellular pH, pOCs, and mOCs can be distinguished. Recently, a pH-sensitive cerium (Ce) nanoparticle (CNS) was synthesized for osteoclast-targeted delivery with alendronate conjugation.^340^ By altering the surface Ce^3+^: Ce^4+^ ratio, the oxidative enzyme activity of nanoparticles could be sensitively triggered at pH 3–4, which is consistent with the microenvironment of bone resorption lacunae, and the cerium particle would decrease the viability of mOCs by over accumulating intracellular oxygen species and over-enhancing calcium oscillation, leading to DNA damage-induced cell cycle arrest and apoptosis. The alendronate moiety enables the delivery of the nanoparticle to the hydroxyapatite. In vitro time-dependent cytoskeleton and focal adhesion staining assays showed that the mOC formation peak, TRAP activity, actin ring formation, and ATPase H^+^ Transporting V0 Subunit D2 expression (ATP6v0d2, a vital proton pump for extracellular acidification and cell-cell fusion), were brought forward from 120 to 72 h at a 100 μg/ml dose, with a decrease of them followed at 120 h. In addition, the early cell apoptotic rate detected by flow cytometry analysis was significantly increased at 72 and 120 h compared to the control group, while the mononuclear pOC number was not altered. Administration of it in OVX mice attenuated bone loss in a 5-week time with an overall anabolic effect on BMD, thickness and bone volume fraction of trabecular and cortical bone. In contrast to the non-selective alendronate group, higher PDGF-BB and CD31^hi^Emcn^hi^ cell expression was detected, which further validated mOC selectivity and indicated the feasibility of mOC-targeted therapy for retaining pOC-induced angiogenesis enhancement.

Similarly, membrane expression markers that distinguish osteoclasts from their precursors can also be specific targeting sites. Calcitonin gene-related peptide (CGRP) is expressed on the surface of monocytes in the early stages of osteoclasts,^341^ while TRAP is a specific protein expressed by mOCs that attach to the bone resorption lacuna.^342^ Zhang et al. constructed two nanoparticles targeting different stages of osteoclasts: one connected with the calcitonin gene-related peptide receptor (CGRPR) and the other attached with specific TRAP peptides. Both nanoparticles were modified with CD44-binding hyaluronic acid (HA), which further fostered delivery to the CD44-rich bone marrow.^343^ The targeting ability of both nanoparticles on bone resorption areas was validated by bone tissue sectioning and small animal biopsy.

In addition to targeting specific membrane markers, cell membrane coating technology has been applied as a promising strategy to evade immune elimination and target homologous cells in nanoparticle-based drug delivery and imaging.^344^ By camouflaging pOC-derived membranes on a reactive oxygen species-responsive cationic polymer containing siRNA against circular RNA BBS9 (circBBS9), a conserved circRNA highly expressed in pOCs promoting multinucleation under RANKL stimuli via the circBBS9/miR-423-3p/TRAF6 axis, Wang et al. synthesized a pOC-targeted nanoparticle.^345^ Administration of it in OVX mice showed a more prominent therapeutic effect in reversing bone mass and microstructure without obvious organ damage compared to bare particles and macrophage membrane-coated particles, which indicated its spatiotemporally targeting ability. Of note, the negative membrane coating enhanced the delivery efficiency of the particles by fusogenically internalization, decreasing endocytosis- and lysosomal degeneration-derived elimination. Moreover, inhibition of the circBBS9/miR-423-3p/TRAF6 axis did not induce an obvious impair of mOC’s resorption ability, which helps preserve physiological osteolytic function for more precise regulation.

#### Osteoblasts

Despite the effect of DSS peptides above as targeting ligands, the accurate binding site of them is the bone formation surface rather than the osteoblasts themselves. Using a phage display technique, Sun et al. identified peptide Ser-Asp-Ser-Asp (SDSSD), which had a binding affinity to periostin (also known as osteoblast-specific factor 2, OSF-2), thus targeting osteoblasts in a specific ligand-receptor specific manner.^346^ Attachment of it to PU nanomicelles containing anti-miR-214 (miR-214 inhibits osteogenic activities by targeting activating transcription factor 4 (ATF4) and enhancing resorption activities via PTEN) prominently improved bone formation and microstructure in OVX mice. In another research by Cui et al., engineered exosomes containing siShn3 were delivered to the skeleton with conjugation of SDSSD peptides, and administration of the complex increased SLIT3 production and facilitated type H vascularization in OVX mice.^347^ In addition, using Cell-SELEX, Liang et al. screened aptamer CH6 as an osteoblast-binding ligand with minimal hepatocyte and peripheral blood mononuclear cell accumulation capacity.^19^ Attachment of it to lipid nanoparticles encapsulating Plekho1 siRNA (CH6-LNPs-siRNA) showed much higher cellular uptake and bone distribution with lower cytotoxicity compared with LNPs-siRNA groups in vitro and in vivo. The promotion of bone formation, bone microstructure, and mechanical properties occurred in both osteopenic and healthy rodents after administration. The knockdown of Plekho1 expression also increased sequentially in the CH6-LNPs-siRNA, LNPs-siRNA, and free-siRNA groups, which further validated its delivery efficiency.

In their fore-and-aft studies, a comparison of delivery efficiency between dioleoyl trimethylammonium propane (DOTAP)-based cationic liposomes attached to DSS with the same siRNA encapsulated in them and CH6-LNPs-siRNA was also conducted.^19,297^ Compared with DSS6-liposome-siRNA, CH6-LNPs-siRNA achieved better gene silencing and bone anabolic effects. The difference should be a result of the targeting sites and RNA vector. DSS_6_ mainly targets their less crystallized hydroxyapatite where bone formation proceeds, while aptamer CH6 directly targets osteoblasts and avoids affecting other cells close to the bone-forming surface, such as endothelial cells and lymphocytes. Additionally, a lower increase in average vector diameter by CH6 conjugation compared with DSS conjugation and high PEG shielding on LNPs possessed CH6-LNPs with higher siRNA encapsulation efficiency, less loss induced by the mononuclear phagocyte system and less detrimental hepar and spleen accumulation.

#### BMSCs

**Fig. 7:**
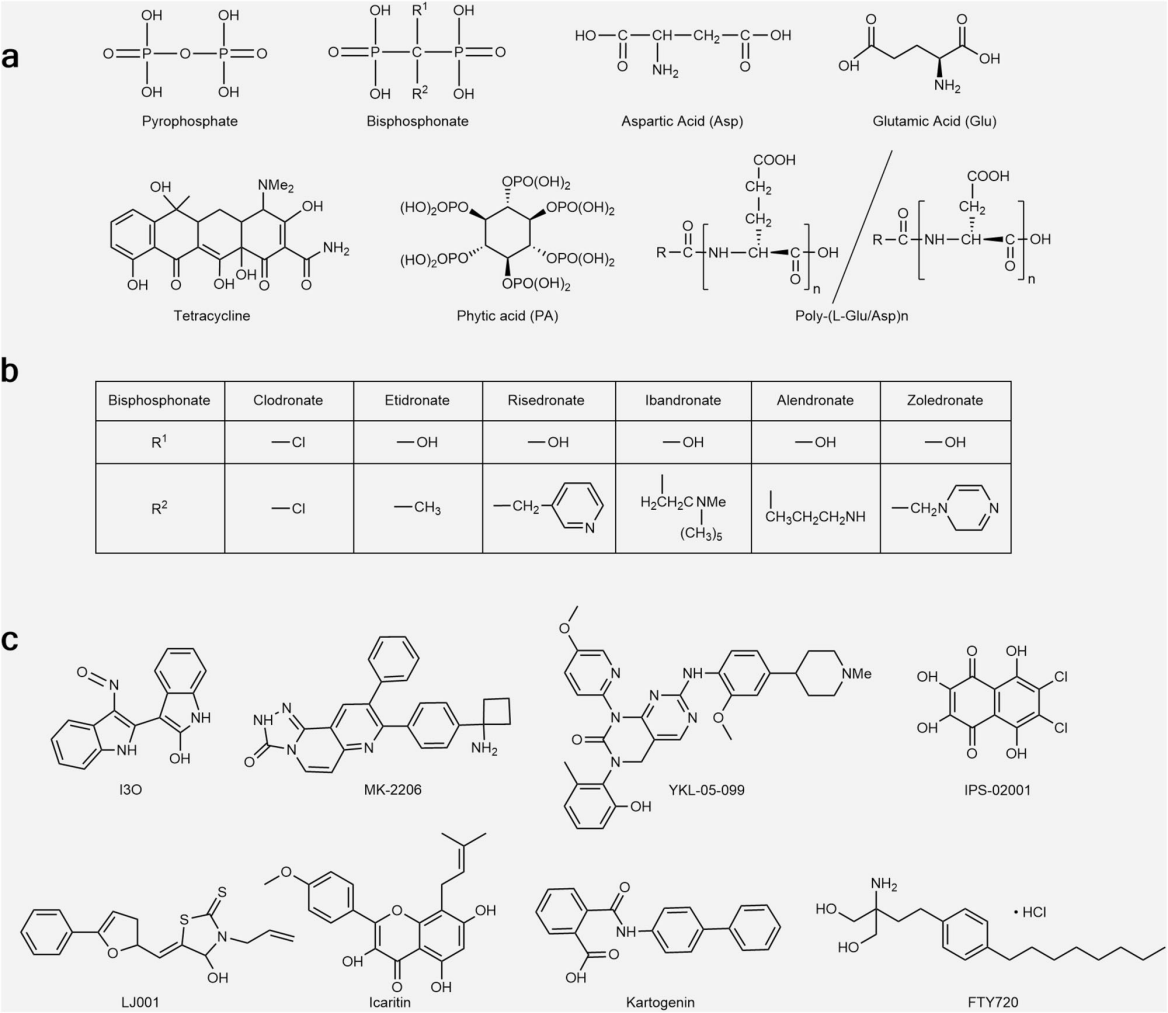
**a** Structures of hydroxyapatite-targeted ligands. **b** R1 and R2 group of different bisphosphonates **c** Structure of small molecule agents mentioned in the text

Apart from delivery via CXCL12/CXCR axis-mediated homing, modification of other BMSC-targeting ligands can also endow vectors with the homing ability and BMSC affinity. In Luo et al.’s research, bone marrow stromal cell-derived exosomes (STExos) were modified with a BMSC-specific aptamer on the surface, and intravenous injection of the STExo-aptamer complex improved bone mass in OVX mice.^348^ Similarly, an MSC-binding peptide EPLQLKM (E7) identified by phage display^349^ was fused to the exosomal membrane protein Lamp 2b to form BMSC-targeted exosomes with kartogenin (KGN, a small molecule that induces differentiation of synovial fluid-derived mesenchymal stem cells toward chondrocytes) (Fig. 7c) encapsulated in.^350^ Both oral administration and intra-articular injection of E7-Exo/KGN manifested more pronounced therapeutic effects in an RA rat model than Exo/KGN and KGN alone. Likewise, the MSC-targeting peptide VTAMEPGQ was proven to possess the same delivery effect.^351,352^

## Clinical application of bone remodeling-targeted therapy

Currently, numerous drugs have been approved by the FDA for bone diseases (Table 3); however, a large proportion of them, especially those for arthritis and bone tumor therapy, still lack bone specificity, while bone-targeted agents are primarily applied in bone metabolism disorders, such as osteoporosis and osteogenesis imperfecta. With the deepening knowledge of bone remodeling biology, preclinical evidence has shown that abnormal bone remodeling can contribute to the genesis or progression of arthritis and bone tumors, while restoring normal bone remodeling could benefit their treatment or prevention. In addition, novel therapeutic agents with promise in better bone remodeling regulation have also been exploited in clinical trials for bone metabolic diseases. Here, we summarized recent advances in the clinical application of bone remodeling-targeted therapy (Table 4).

**Table 3:** FDA-approved drugs for bone diseases

Bone disease	Action mechanism	Drug	Drug type	Year of FDA approval	Therapy type
Osteoporosis	ERs agonist	Estrogens, Esterified/Conjugated	Chemical	1964/1942	Hormone replacement therapy
		Next-generation transdermal estrogen		2013	
		Estradiol/Norethindrone Acetate		2000	
		Estrone sodium sulfate/Sodium equilin sulfate		2009	
		Sodium estrone sulfate/Sodium equilin sulfate/Medroxyprogesterone acetate		2009	
		Conjugated Estrogens/Medroxyprogesterone Acetate		1995	
	SERM	Raloxifene hydrochloride		1997	Bone-targeted therapy
		Bazedoxifene/Conjugated estrogens		2013	
	FDPS inhibitor	Zoledronic acid		2008	
		Risedronate sodium		1998	
		Alendronate Sodium/Cholecalciferol		2005	
		Alendronate sodium		1995	
	Osteoclast inhibitor	Ibandronate		2003	
	PTH1R agonist	Abaloparatide	Recombinant polypeptide	2017	
		Teriparatide		2002	
	SOST inhibitor	Romosozumab	Monoclonal antibody	2019	
	RANKL inhibitor	Denosumab		2010	
Osteoarthritis and rheumatoid arthritis	COX inhibitor	Indomethacin	Chemical	1984	Disease-modifying therapy
		Fenoprofen Calcium		1976	
		Naproxen		1976	
		Naproxen sodium		1980	
		Piroxicam		1982	
		Diclofenac sodium		2007	
		Oxaprozin		1992	
		Diclofenac sodium/Misoprostol		1997	
		Celecoxib		1998	
		Meloxicam		2000 (OA)/ 2004 (RA)	
		Esomeprazole Magnesium/Naproxen		2010	
		Famotidine/Ibuprofen		2011	
	GR agonist	Triamcinolone Acetonide		1960	
		Methylprednisolone Acetate		1959	
		Triamcinolone Acetonide		1960	
Osteoarthritis	IL-17A inhibitor	Secukinumab	Monoclonal antibody	2021	
	Sodium hyaluronate	Cross-linked sodium hyaluronate injection	Biological drugs	2007	
Rheumatoid arthritis	DHFR inhibitor	Methotrexate sodium	Chemical	1953	
		Methotrexate		2019	
	JAK inhibitor	Upadacitinib		2019	
		Baricitinib		2018	
	GR agonist	Methylprednisolone acetate		1959	
	DHODH inhibitor	Leflunomide		1998	
	IKK inhibitor	Auranofin		1985	
	CTGF inhibitor	Penicillamine		1970	
	Immunosuppressant	Azathioprine		1968	
	Leukotriene synthesis inhibitor	Sulfasalazine		1950	
	CD20 inhibitor	Rituximab	Monoclonal antibody	2006	
	IL-6RA antagonist	Sarilumab		2017	
		Tocilizumab		2010	
	TNF-α inhibitor	Golimumab		2009	
		Certolizumab Pegol		2009	
		Adalimumab		2002	
		Infliximab		1999	
		Etanercept	Fusion protein	1998	
	CD86 and CD80 regulator	Abatacept		2005	
	IL1R1 antagonist	Anakinra	Interleukin	2001	
	Melanocortin receptor agonist	Corticotropin	Synthetic polypeptide	1950	
Bone tumor	FDPS inhibitor	Pamidronate disodium	Chemical	2002	Bone targeted therapy
		Zoledronic acid		2002	
	TYMS inhibitor	Levofolinate calcium		2008	
	DHFR inhibitor	Methotrexate sodium		1959	
	DNA inhibitor	RADIUM RA-223 DICHLORIDE		2013	
	RANKL inhibitor	Denosumab	Monoclonal antibody	2010	
Paget disease of bone	FDPS inhibitor	Zoledronic acid	Chemical	2007	
		Risedronate sodium		1998	
	Osteoclast inhibitor	Etidronate disodium		1977	
	CTR agonist	Calcitonin salmon	Synthetic polypeptide	1975	
Hypophosphatasia	Recombinant ALP	asfotase alfa	Peptide-drug conjugate	2015	

*ER* estrogen receptor, *SERM* selective estrogen receptor modulators, *FDPS* farnesyl diphosphate synthase, *PTH1R* type 1 parathyroid hormone receptor, *RANKL* receptor activator of nuclear factor-kB ligand, *COX* cyclooxygenase, *GR* glucocorticoid receptor, *DHFR* dihydrofolate reductase, *JAK* Janus kinase, *DHODH* dihydroorotate dehydrogenase, *IKK* I kappa B kinase, *CTGF* connective tissue growth factor, *TYMS* thymidylate synthetase, *CTR* calcitonin receptor, *ALP* alkaline phosphatase

**Table 4:** Recent clinical trials targeting bone remodeling to treat bone diseases

Individual	Agent	Target	Type	Source	Enrollment	Sponsor	Phase	Status
**Osteoporosis**								
Postmenopausal osteoporosis	Denosumab	RANKL	Monoclonal antibody	NCT00089791	7808	Amgen	Phase III	Completed
Glucocorticoid-induced Osteoporosis				NCT03164928	24	Amgen	Phase III	Active, not recruiting
Osteoporosis associated to systemic mastocytosis				NCT03401060	24	Assistance Publique - Hôpitaux de Paris	Phase III	Active, not recruiting
Postmenopausal osteoporosis	Romosozumab (versus Alendronate	Sclerostin		NCT01588509	60	Amgen	Phase I	Completed
Chinese postmenopausal osteoporosis	Romosozumab			NCT05067335	564	UCB Biopharma SRL	Phase III	Active, not recruiting
Glucocorticoid-induced Osteoporosis	Romosozumab (versus Denosumab)			NCT04091243	72	Tuen Mun Hospital	Phase IV	Active, not recruiting
Women with low bone mineral density	Blosozumab (LY2541546)			NCT01144377	154	Eli Lilly and Company	Phase II	Completed
Postmenopausal osteoporosis	SHR-1222			NCT04435158	107	Jiangsu HengRui Medicine Co., Ltd.	Phase I	Active, not recruiting
	MK-0822	Cathepsin K	Small-molecule inhibitor	NCT00529373	16071	Merck Sharp & Dohme LLC	Phase III	Completed
	Teriparatide	PTH1R	Recombinant polypeptide	NCT01709110	1366	Eli Lilly and Company	Phase IV	Completed
	Lactobacillus Reuteri	Gut-bone axis	Probiotics	NCT04169789	239	Sahlgrenska University Hospital, Sweden	Not applicable	Completed
	Lactobacillus Acidophilus			NCT05332626	60	Poznan University of Life Sciences		Enrolling by invitation
Motor-complete spinal cord injury-induced osteoporosis	Rosuvastatin Calcium	Hepatic hydroxymethyl-glutaryl coenzyme A	Small-molecule inhibitor	NCT03113994	8	Dr. B. Catharine. Craven	Phase II	Active, not recruiting
	Simvastatin			NCT02946424	20	Craig Hospital	Phase II	Active, not recruiting
Postmenopausal osteoporosis	Atorvastatin			NCT02342015	20	University of Padova	Phase IV	Completed
	MK-0429	α5β3		NCT00533650	227	Merck Sharp & Dohme LLC	Phase II	Completed
Osteoporosis	Fucosylated BMSCs	Bone marrow	Cell	NCT02566655	10	Red de Terapia Celular	Phase I	Completed
Osteopenia secondary to glucocorticoids	LLP2A-Ale	MSC	PDB	NCT03197623	58	Nancy E. Lane, MD	Phase I	Completed
Postmenopausal osteoporosis	Quercetin	Senescent cell	Natural senolytic	NCT05371340	33	Kennesaw State University	Not applicable	Completed
Healthy elderly women	Dasatinib, Quercetin, Fisetin		Senolytics	NCT04313634	120	Sundeep Khosla, M.D.	Phase II	Recruiting
**Osteoarthritis (OA)**								
Patients scheduled for total knee replacement	LNA043	Integrin α5β1	Small-molecule drug	NCT02491281	28	Novartis Pharmaceuticals	Phase I	Completed
Knee OA				NCT04864392	550		Phase II	Recruiting
	MIV-711	Cathepsin K		NCT03037489	50	Medivir	Phase II	Completed
				NCT02705625	244			
	TGF-β/SMAD signaling pathway level			NCT05218122	340	Dongzhimen Hospital, Beijing	Observational trial	Recruiting
	Tanezumab	NGF	Monoclonal antibody	NCT00830063	832	Pfizer	Phase III	Completed
	Quercetin with/without Fisetin	Senescent cell	Natural senolytic	NCT05276895	60	Assiut University	Not applicable	Not yet recruiting
	Fisetin			NCT05482672	120	Cale Jacobs, PhD	Phase II/ III	Not yet recruiting
				NCT04210986	75	Steadman Philippon Research Institute	Phase I/II	Active, not recruiting
OA of interphalangeal finger joints	Denosumab	RANKL	Monoclonal antibody	NCT02771860	100	University Hospital, Ghent	Phase II	Completed
OA of the hip or knee	TPX-100	Subchondral matrix	Small peptide	NCT02528188	3021	OrthoTrophix, Inc	Phase II	Completed
				NCT02837900	14		Phase II	Completed
**Rheumatoid arthritis (RA)**								
RA	Denosumab	RANKL	Monoclonal antibody	NCT01973569	679	Daiichi Sankyo, Inc.	Phase III	Completed
				NCT00095498	227	Amgen	Phase II	Completed
Juvenile idiopathic arthritis	Sema4A level			NCT05534347	300	Assistance Publique - Hôpitaux de Paris	Observational trial	Not yet recruiting
**Osteogenesis Imperfecta (OI)**								
OI	Romosozumab	Sclerostin	Monoclonal antibody	NCT04545554	25	Amgen	Phase I	Active, not recruiting
Type I, III or IV OI	Setrusumab (BPS804)			NCT03118570	112	Ultragenyx Pharmaceutical Inc	Phase II	Completed
OI				NCT05125809	219	Ultragenyx Pharmaceutical Inc	Phase II/III	Recruiting
	Fresolimumab	TGF-β		NCT03064074	11	Baylor College of Medicine	Phase I	Completed
Adult OI	SAR439459			NCT05231668	24	Sanofi	Phase I	Recruiting
OI	Bone marrow-derived mesenchymal stromal cells	N.A.	Cell	NCT05559801	12	Emory University	Phase I/II	Not yet recruiting
	Bone marrow-derived mesenchymal stromal cells			NCT03706482	18	Karolinska Institutet	Phase I/II	Active, not recruiting
**Nontraumatic osteonecrosis**								
Osteonecrosis of femoral head	Zoledronic Acid	Osteoclast	BP	NCT00939900	110	Seoul National University Bundang Hospital	Phase III	Completed
Nontraumatic osteonecrosis	RAB001	MSC	PDB	CTR20222771	n.a.	RabPharma	Phase I	Not yet recruiting
Nontraumatic osteonecrosis of the knee	Ibandronate	Osteoclast	BP	NCT00532220	30	University Hospital, Basel, Switzerland	Phase III	Completed
**Osteosarcoma**								
Recurrent or refractory osteosarcoma	Denosumab	RANKL	Monoclonal antibody	NCT02470091	56	Children’s Oncology Group	Phase II	Active, not recruiting
Recurrent, refractory, or progressive pulmonary metastatic osteosarcoma	Natalizumab	α4-integrin		NCT03811886	20	Case Comprehensive Cancer Center	Phase I/II	Recruiting
Osteosarcoma	ALMB-0168	Connexin 43		NCT04886765	238	AlaMab Therapeutics (Shanghai) Inc.	Phase I/II	Not yet recruiting
Recurrent, relapsed, or refractory solid tumors including osteosarcoma	Pepinemab	Sema 4D		NCT03320330	26	Children’s Oncology Group	Phase I/II	Active, not recruiting
Osteosarcoma	Chemotherapy (with or without zoledronic acid)	Osteoclast	BP	NCT00470223	318	UNICANCER	Phase III	Active, not recruiting
**Bone metastasis**								
Hormone refractory prostate cancer	Denosumab	RANKL	Monoclonal antibody	NCT00286091	1435	Amgen	Phase III	Completed
Unresectable or metastatic melanoma	Denosumab in combination with immune checkpoint inhibitors			NCT03161756	72	Melanoma and Skin Cancer Trials Limited	Phase I/II	Active, not recruiting
Lung cancer with bone metastases	Denosumab with Nivolumab			NCT03669523	82	Centre Hospitalier Annecy Genevois	Phase II	Active, not recruiting
**Paget’s Disease of Bone**								
Pathophysiology of Paget’s Disease of Bone	N.A.	N.A.	N.A.	NCT02802384	11	Johns Hopkins University	Observational trial	Active, not recruiting

*BP* bisphosphonate, *PDC* peptide-drug conjugate, *MSC* mesenchymal stem cell, *NGF* nerve growth factor, *Sema* semaphorin, *TGF-β* transforming growth factor-β

### Osteoporosis

As a chronic skeletal disease characterized by imbalanced bone remodeling and deteriorated bone microstructure, osteoporosis can cause a high risk of fragile fractures in the spine, hip, wrist, et al., and has become a significant global health problem due to its prevalence worldwide and the aging of the population. The primary focus of osteoporosis-related therapy is to restore bone homeostasis and prevent fractures. Nevertheless, owing to its multiple and overlapping pathogeneses,^353^ it is challenging to develop a universal etiological therapy besides the basic vitamin D and calcium supplements. Generally, therapeutic drugs for osteoporosis can be classified into anti-resorption agents, bone anabolic agents, and agents with dual effects, with several classes of them approved by the FDA (Table 3). The main indication of these established drugs remains postmenopausal osteoporosis, although several expanding access of them has entered several trials. As most of them have been discussed in the preceding sections, here we mainly discuss recent trials of developing novel agents for osteoporosis.

Inhibiting bone resorption without an attendant suppression in bone formation is still unattainable for all currently approved antiresorptive agents for osteoporosis (bisphosphonates, denosumab, estrogen, and SERMs).^6^ How to decrease resorption activities while retaining positive signaling from osteoclasts such as Ephrin-Eph signaling,^194^ WNT-10b, and S1P,^83^ is a key obstacle for improving anti-resorption therapies. Targeting molecules in the resorption lacuna, such as cathepsin K, was considered an alternative strategy. In preclinical studies, cathepsin K inhibitors preserved the normal morphology of osteoclasts with a slight increase in their number on the bone surface, in contrast to the denosumab-induced decrease in number and bisphosphonate-induced apoptotic and giant, hypermultinucleated morphology alteration,^354,355^ which indicated a lesser influence on physiologic bone resorption. Unfortunately, with the drop by Merck & Co. on osteoporosis drug odanacatib (MK-5442, a cathepsin K inhibitor) due to an increased risk of cardiovascular events,^356^ the development of novel anti-resorption agents targeting the resorption lacuna molecules seems to have reached a bottleneck. As “coworkers” with cathepsin K in the ruffled border of OCs, chloride channel-7 (ClC-7) was also proven to be a therapeutic target for osteoporosis.^357,358^ Nevertheless, no subsequent studies and trials were conducted, probably due to the risk of a severe osteopetrosis phenotype or neurodegeneration induced by ClC-7 chloride channel loss in preclinical models.^359,360^ As a significant factor mediating osteoclasts to the resorption surface, α5β3 has been proven as a promising target in preclinical studies.^361^ In addition, α5β3 deletion would not cause a severe osteopetrosis phenotype as ClC-7 deletion,^362^ which indicated its translation potential. A previous trial showed that administration of MK-0429, an α5β3 inhibitor, was well-tolerated and showed apparent increases in lumbar spine BMD in postmenopausal osteoporotic women.^363^ Nevertheless, the increase in hip BMD required a higher dose.

As previously stated, recent preclinical studies have highlighted the significance of the gut-bone axis.^102^ Several trials have been conducted to evaluate the influence of probiotic administration in postmenopausal women with osteoporosis. Similarly, the association between statins and a decreased risk of osteoporosis has also triggered some trials (Table 4).^364^ Despite the therapeutic effect of stem cell homing in numerous preclinical studies,^365,366^ only two trials have been completed involving the intravenous infusion of fucosylated autologous BMSCs in patients with established osteoporosis and low-impact fractures, and intravenous infusion of LLP2A-Ale to promote BMSC homing in patients with glucocorticoid-induced osteopenia. However, the results from these trials have yet to be posted. The limited progress in stem cell therapy-related trials may be attributed to the inconvenience of cell preparation, the risk of tumorigenesis and thrombosis, and their limited delivery efficiency in vivo.^367^

Of note, antisenescence agents, also known as senolytics, are being studied as a potential treatment for age-related skeletal diseases by decreasing the senescence phenotype of bone and cartilage cells.^368^ In preclinical studies, senolytics such as dasatinib (an FDA-approved tyrosine kinase inhibitor),^369^ quercetin, and fisetin (natural senolytics derived from fruits and vegetables)^370,371^ have shown promising effects in decreasing bone resorption and improving trabecular and cortical bone microarchitecture in aged mice. In addition, senolytics were also shown to attenuate the progression of osteoarthritis and age-dependent intervertebral disc degeneration in mice,^372,373^ with no discernible impact on the proliferating, quiescent, and differentiated bone cells.^374^ A combination of dasatinib and natural senolytics may maximize the therapeutic effect in the skeleton, owing to their different preferences for progenitor cell lineage,^375^ while using natural senolytics alone may result in fewer side effects. A few trials have recently been conducted to assess the in vivo effect of natural senolytics in osteoporosis and osteoarthritis patients (Table 4).

### Bone tumor and metastasis

#### Osteosarcoma

As a rare skeletal malignancy, osteosarcoma primarily implicates children and adolescents (4.4 cases per million individuals) and adults over the age of 65 (4.2 cases per million individuals). It commonly arises in weight-bearing long bones (43% in distal femur, 23% in proximal tibia, and 10% in humerus proximal) with a 5-year survival rate of ~70% but drastically decreases to less than 30% after metastasis.^376^ Unfortunately, little progress has been made in improving osteosarcoma survival since the establishment of standard surgery and induction and consolidation chemotherapy in the 1980s.^377^ As mentioned above, targeting the initiating genes in osteosarcoma is challenging owing to high heterogeneity. An alternative strategy is to develop drugs targeting signaling pathways upstream/downstream of the initiating genes. Receptor tyrosine kinases (RTKs) are key factors associated with cell viability, proliferation, survival et al., and abnormal activation of several RTKs, including vascular endothelial growth factor receptors (VEGFRs), FGFRs, rearranged during transfection (RET), epidermal growth factor receptors (EGFRs), insulin-like growth factor receptors (IGFRs), PDGFRs, et al., has been proven to drive osteosarcoma genesis.^378^ Small-molecule oral multiple tyrosine kinase inhibitor (MTKI) agents, especially VEGFRs/RET-targeted agents, have shown promising effects on progression-free survival in patients with relapsed and unresectable high-grade osteosarcoma and metastatic osteosarcoma. In addition, osteosarcoma cell surficial receptors, such as glycoprotein nonmelanoma protein B (GPNMB, also known as osteoactivin), and leucine-rich repeat containing protein 15 (LRRC15), were also proven as promising targets with a few trials conducted.^379^

The lack of effective consolidation therapy after standard chemotherapy has raised interest in investigating whether targeting bone remodeling can generate additional benefits in osteosarcoma therapy, noticing denosumab’s approval for giant-cell tumor of bone (GCT). However, unlike GCT and osteoporosis, the osteosarcoma microenvironment is heterogeneous, with a predominantly osteoblastic, osteolytic, or mixed lytic/proliferative skeleton change.^380^ In addition, whether the improvement of skeletal-related events and decreasing metastasis rate can be coordinated remains indistinct, thus making it challenging to apply additional bone remodeling therapy, with some conflicting preclinical studies. For instance, bisphosphonates were reported to alleviate osteosarcoma-induced osteolysis by suppressing MCP-1 and RANKL expression in osteosarcoma cells.^381^ However, it was also shown to promote osteosarcoma lung metastasis due to osteoclast loss.^382^ An interim analysis of an ongoing phase 3 trial also showed a slight increase induced by BPs in the recurrence and metastasis rates.^383^ In contrast, oral administration of the RANKL inhibitor AS2676293 was reported to suppress bone metastasis,^384^ and RANKL supplementation may attenuate the deteriorated trabecular structure in some cases by reactivating osteoclastogenesis. These inconsistent results confirm the complex bone remodeling induced by osteosarcoma, and preclinical studies may not provide sufficient efficacy to determine whether additional bone remodeling agents can generate a benefit. Fortunately, a few related trials have been conducted for further exploitation (Table 4).

#### Bone metastasis

As a prevalent biological activity occurring in 70 to 80% of aggressive cancers, bone metastasis can cause unbearable pain, hypercalcemia, spinal cord compression, and pathologic fractures, which greatly impair the quality of life among affected patients.^385^ The underlying mechanisms of bone metastasis are multifactorial and involve bidirectional interactions between tumor cells and bone and bone marrow microenvironment, as postulated by the “seed and soil” theory proposed by Stephen Paget.^386^ Three bone-targeted drugs, zoledronic acid, pamidronate disodium, and denosumab, have been approved by the FDA for bone metastasis induced skeletal-related events, including lytic lesions and fragile fractures.^387^ Among them, denosumab was found to exhibit greater compliance and longer persistence in patients with bone metastasis than zoledronic acid, as shown in a long-term treatment study.^387^ A superior delay or prevention of skeletal-related events (SREs) by denosumab compared to zoledronic acid was also validated by several phase 3 trials of bone metastasis patients with advanced breast cancer^388^ and castration-resistant prostate cancer.^389^ In patients with multiple myeloma or other advanced cancers, excluding breast and prostate cancer, denosumab showed a noninferior (trending to superiority) effect compared to zoledronic acid.^390^ In addition, a phase 3 trial of 1435 individuals demonstrated that denosumab treatment could delay bone metastasis in men with castration-resistant prostate cancer,^391^ as shown by zoledronic acid treatment in patients with stage IIIA and IIIB non-small cell lung cancer.^392^ These results indicated that denosumab and zoledronic acid not only prevent SREs but also might prevent bone metastasis.

Recently, RANK/RANKL pathway-mediated immune regulation has been emphasized in preclinical studies of cancer treatment. Inhibition of RANK/RANKL signaling by denosumab induced an orchestrated antitumor immune response increasing CD8^+^ T-cell-mediated tumor cytotoxicity and decreasing neutrophil-mediated immunosuppression in breast cancer,^393^ which indicates that RANKL suppression may generate a synergistic therapeutic effect in primary and metastatic lesions. It was further discovered that a higher serum RANKL/OPG level is a prognostic factor associated with breast cancer metastasis.^394^ Currently, two ongoing trials are investigating the potential of denosumab in combination with chemotherapy to promote tumor inhibition (Table 4).

### Nontraumatic osteonecrosis

Nontraumatic osteonecrosis (NTON) comprises a class of prevalent but refractory bone osteonecrosis, such as nontraumatic osteonecrosis of the femoral head (ONFH) and nontraumatic knee necrosis (ONK). The impaired blood supply and bone remodeling, induced by glucocorticoid administration, alcohol abuse hyperlipidaemia, blood dyscrasias, and systematic inflammatory diseases, are the underlying pathogenesis that causes bone cell death and bone microstructure collapse, leading to a decreased life quality and subsequent decompression or replacement surgery demands.^395^ Unfortunately, no bone remodeling drugs have been approved for it. Although bisphosphonate has shown promise in preclinical studies^396,397^ and in a trial treating bone marrow edema in ONK,^398^ it did not demonstrate additional benefits in preventing collapse and reducing the need for total hip arthroplasty in a phase 3 trial enrolling 110 participants.^399^ In addition, prolonged bisphosphonate usage may generate a risk of mandibular osteonecrosis.^400^

Mechanistically, the pathogenesis of NTON involves abnormal crosstalk between endothelial cells and bone cells, manifested in a decreased serum CXCL12/SDF-1 level,^401^ which is crucial for stem cell and pOC homing and angiogenesis.^367,402,403^ Thus, stem cell therapy has been exploited in NTON treatment. In a multicentric, 5-year follow-up trial, administration of autologous, expanded, bone marrow-derived mesenchymal stromal cells safely healed OFNH.^404^ Furthermore, expanded autologous mesenchymal stem cells fixed in grafted allogenic bone tissue also exhibited therapeutic effects.^405^ In addition to autologous stem cell infusion, mobilizing endogenous stem cell homing has also been investigated to treat OFNH. As mentioned above, LLP2A-Ale can form new bone and increase bone strength by directing MSCs to the bone marrow in xenotransplantation and immunocompetent mice.^252^ A phase 1 trial was subsequently conducted to assess the safety and tolerability of intravenous LLP2A-Ale administration in adult men and women with osteopenia secondary to corticosteroids (Table 4). Rab001, a BMSC-homing compound consisting of a peptidomimetic ligand with integrin α4β1 (VLA-4) affinity and a bisphosphonate motif, was shown to promote bone mass with increased CD31^HI^EMCN^HI^ vessels and attenuated osteonecrosis in a glucocorticoid-induced osteonecrosis mouse model,^406^ demonstrating the therapeutic potential of BMSC homing promotion agents. Currently, Rab001 has been approved by the National Medical Products Administration (NMPA) for a phase 1 trial (CTR20222771).

### Rare skeletal diseases

#### Paget’s disease of bone

As a chronic bone disease characterized by a high focal turnover rate, Paget’s disease of bone (PDB) mainly occurs in middle-aged or elderly individuals, manifested in a more enormous, more sclerotic, yet vulnerable skeleton with a deterioration risk.^407^ Despite significant advances in knowledge of its pathology, pathophysiology, and epidemiology since its first report in 1876,^408^ further elucidation is required regarding the decreased incidence and severity, the role of environmental and genetic factors, and the mechanism of its abnormal bone remodeling.^409^ From the initial osteolytic lesions induced by larger osteoclasts with more nuclei,^410^ to the dense but brittle osteogenic lesions accompanied by hypervascularity and high serum ALP levels in the later stage,^410^ the interplay between bone resorption and formation in PDB remains ill-defined. Intriguingly, a similar skeletal phenotype with a high turnover rate and disorganized bone remodeling was observed in Camurati-Engelmann diseases with TGF-β1 mutation and in mice with TGF-β1 overexpression.^411,412^ As a coupling factor of bone remodeling,^413^ TGF-β1 guides BMSCs to the resorption sites, and this process is dependent on osteoclast resorption to activate the inactive TGF-β1 in the bone matrix.^414^ Nevertheless, the mechanism by which osteoclasts trigger such decoupling in PDB remains elusive. An ongoing cross-sectional study investigating osteoclast-derived chemokines in PDB patients may provide more clues into the pathogenesis.

For the treatment of PDB, effective management has been established based on a few agents that can suppress the accelerated bone turnover rate. Calcitonin, a peptide hormone targeting osteoclast surficial receptors, was the first therapeutic agent introduced in 1968 that provides quick pain relief.^415^ However, daily injections and frequent flushing or nausea soon led to the replacement by bisphosphonates owing to a more convenient administration route, more potent suppression of high bone turnover, and long-acting effects owing to their tropism to the bone mineral.^416^ With more bisphosphonate agents introduced in the clinic, various oral or intravenous administration regimens have been established for PDB treatment. Among them, zoledronate therapy is the most effective with a single intravenous dose leading to a 6-month normalization of ALP levels in 96% of patients in a phase 3 trial compared with the 74.3% rate of 60 days oral risedronate administration.^417^ A follow-up study found that 64% of individuals showed some loss of zoledronate effect after 9 years of the single dose, yet only 14% detected biochemical relapse.^418^ Owing to its considerable effect, trials investigating other bone-targeted drugs in PDB treatment were not further conducted on a large scale. Of note, although a relapse of PDB can be successfully treated with a 5-milligram infusion of zoledronic acid (NCT00740129), over administration of BPs should be avoided after pain relief for no extra benefit but an increased risk of osteonecrosis.

#### Osteogenesis imperfecta

Despite exhibiting a similar osteopenia phenotype, osteogenesis imperfecta (OI) is distinguished from osteoporosis by its impaired bone mineralization rather than abnormal bone remodeling.^419^ Most OI cases are characterized by mutations in genes encoding type I collagen, such as COL1A1 or COL1A2, with other gene mutations observed in 15–25% of cases.^420^ Five primary clinical forms of OI have been identified by nosology and classification of genetic skeletal disorders.^421^ Unfortunately, gene-specific therapies have yet to be feasible for OI. Currently, bisphosphonates, such as pamidronate, alendronate, and zoledronic acid, are widely administered as supportive therapy to treat OI.^422^ Although the aberrant collagen would still be deposited in the bone matrix, suppressing osteoclast activities could relatively increase bone mass. However, response to BP therapy is not apparent in severe types of OI or adult OI compared to most pediatric patients.^423^ Denosumab, another anti-resorption drug that improves the BMD of OI patients in several small-case studies, was exploited as an alternative.^424,425^ Nevertheless, denosumab therapy was bothered by a prominent risk of hypercalcemia and hypercalciuria.^426^ Two follow-up trials were also terminated owing to high serum calcium safety concerns (NCT03638128 and NCT02352753). Additionally, teriparatide, a bone anabolic PTH analog, has shown similar poor therapeutic responses in the treatment of moderate and severe cases of OI (NCT00131469).^427^

The inefficiency of single anti-resorption or bone formation agents in managing moderate and severe OI has propelled the exploitation of bone remodeling agents with dual effects. Recent studies have underscored the significance of the impairment of WNT and BMP signaling pathways in OI genesis.^428^ Preclinical studies of sclerostin antibodies in the OI mouse model have demonstrated improved skeletal parameters compared to anti-resorption agents.^429,430^ In a recently finished 12-month phase 2b trial enrolling 112 types I, III, and IV adult OI patients (NCT03118570), administration of setrusumab (BPS804), a sclerostin antibody, showed increased lumbar, total body, and femoral neck BMD, bone strength and remodeling effect, and decreased fracture risk. Although it failed to meet the primary endpoint of trabecular volumetric bone mineral density (Tr vBMD) improvement, these results indicate its promising therapeutic potential, and it was granted rare pediatric disease designation for OI in 2020. Meanwhile, romosozumab, an approved sclerostin antibody for osteoporosis, has also entered a phase 1 trial for OI.

In addition to WNT, TGF-β signaling pathway alteration has also been implicated in OI genesis.^431^ Overexpression of TGF-β signaling was found in both recessive (Crtap^−/−^) and dominant (Col1a2^tm1.1Mcbr^) OI mouse models in a preclinical study, and inhibition of TGF-β with antibodies rescued the bone phenotype with enhanced trabecular and cortical bone mass and strength in both forms of OI models.^431^ A recent gene ontology (GO) enrichment assay of bones derived from type I and III OI children by Song et al. also showed that SMAD phosphorylation downstream of BMP was the most significantly upregulated molecular event, and the TGF-β pathway was identified as the key activated upstream regulator by gene set enrichment analysis (GSEA), and ingenuity pathway analysis (IPA).^432^ Hitherto, a phase 1 trial has been completed to evaluate the safety and efficacy of fresolimumab, a TGF-β antibody, in treating moderate-to-severe OI (NCT03064074). The results showed that fresolimumab was well-tolerated and increased the lumbar spine areal bone mineral density of type IV OI patients, whereas that of type III and VIII OI patients was decreased or unchanged, suggesting that the effect of anti-TGF-β therapy may be associated with a specific gene-phenotype. A forthcoming phase 1 trial evaluating the safety and efficacy of SAR439459, another anti-TGF-β monoclonal antibody, in adults with OI, is expected to yield additional insights (Table 4).

### Arthritis

Distinguishing from the skeletal diseases described above, arthritis, including osteoarthritis (OA) and rheumatoid arthritis (RA), is characterized by abnormal inflammatory destruction during joint chondrocytes and cartilage’s extracellular matrix (ECM) remodeling, with or without autoimmune irregulation.^433^ Hitherto, arthritis remains a significant global health problem owing to the increasing morbidity rate and lack of impactful drug therapy.^434–436^ Currently approved drugs such as nonsteroidal anti-inflammatory drugs (NSAIDs), glucocorticoids (GCs), and conventional synthetic disease-modifying anti-rheumatic drugs (csDMARDs) remain ineffective in attenuating disease progression and can cause nonnegligible systemic side effects due to their low cartilage-targeting ability.^437^ Fortunately, with the deepening knowledge of cartilage metabolism and immune and epigenetic regulation, novel targeted drugs, including disease-modifying osteoarthritis drugs (DMOADs), such as nerve growth factor (NGF) inhibitors,^438^ WNT/β-catenin inhibitors,^439^ and biological DMARDs (bDMARDs), such as adalimumab,^440^ or targeted synthetic DMARDs (tsDMARDs) such as tofacitinib,^441^ and baricitinib,^442^ have emerged with promising therapeutic effects in relieving pain and attenuating progression for moderate to severe OA and RA. These advances in OA and RA treatment have been fully reviewed by Yao et al.^443^ and Ding et al.,^444^ respectively. Notably, although these diseases are characterized by inflammation-induced disorders of the intra-articular microenvironment,^445^ abnormal bone remodeling in the subchondral bone and bone marrow environment, including type H vessel invasion, excessive bone resorption and subsequent osteophyte formation, and decreased chondrogenic differentiation of BMSCs, can also contribute to arthritis progression.^446–448^ Preclinical studies have demonstrated the therapeutic potential of targeting the subchondral microenvironment and some trials have also been conducted to investigate their efficacy, which may provide novel insights for arthritis treatment.

The impaired chondrogenic differentiation ability of BMSCs is a critical factor driving cartilage degeneration. Conversely, promoting chondrogenesis has been demonstrated in numerous preclinical studies to benefit cartilage regeneration and ameliorate arthritis progression.^449,450^ By screening 6300 proteins secreted by MSCs, Gerwin et al. identified angiopoietin-like 3 (ANGPTL3) as a potent stimulator of chondrogenesis.^451^ LNA043, a 26-kDa derivative of ANGPTL3, was also synthesized as a novel disease-modifying OA drug candidate by a single anti-proteolysis point mutation in the core carboxy (C)-terminal fibrinogen-like domain of ANGPTL3. Preclinical testing of LNA043 in OA and cartilage injury models showed a prominent effect in the preservation and regeneration of healthy hyaline cartilage by binding with integrin α5β1, the fibronectin receptor on MSCs and chondrocytes, upregulating DKK-1 and frizzled-related protein to decrease WNT and BMP signaling expression, which has been shown in previous studies to possess cartilage anabolic effects.^452^ In addition, a phase 1 trial of LNA043 in OA patients (NCT02491281) showed well-tolerated and safe administration, rapid systemic distribution, effective cartilage penetration, and lingering of LNA043, which indicated its clinical translation prospects. Currently, a phase 2 trial is ongoing for further assessment (NCT04864392).

In addition to promoting chondrogenic differentiation of BMSCs as a supplement at the source, attenuating excessive type H vessel invasion and osteoclast-mediated erosion of subchondral bone and cartilage are also potential targets for arthritis. During arthritis genesis, apart from excessive osteoclast activities induced by upregulated factors such as IL-1β, TNF-α, IL-6, and HMGB1, in chondrocytes,^453,454^ TGF-β-mediated type H vessel invasion and Th17 differentiation in the subchondral microenvironment also contribute to arthritis progression.^455–457^ Intraperitoneal injection of TGF-β1R inhibitors attenuated chondrocyte apoptosis and cartilage degradation with decreased type H angiogenesis and osteoclast activities in subchondral bone of OA rat models. In addition, halofuginone, a small molecule derivative of febrifugine that has been granted orphan drug status for scleroderma and Duchenne muscular dystrophy, was proven to alleviate osteoarthritis progression with decreased type H vessel invasion in subchondral bone by suppressing SMAD2/3-dependent TGF-β signaling in BMSCs.^458,459^ These results indicate the therapeutic potential of targeting TGF-β in the subchondral bone to treat arthritis. Currently, a cross-sectional trial without intervention is ongoing to assess TGF-β/SMAD signaling pathway expression alterations in OA patients (NCT05218122). Recently, Sema4A was identified as a potent factor in RA in preclinical studies.^460^ Under stimuli by TNF-α et al., EC-derived Sema4A-Plexin-D1 signaling can promote type H angiogenesis, Th17 differentiation, and the expression of inflammatory factors in synovial cells.^461,462^ A trial assessing whether Sema4A can be an angiogenic biomarker in juvenile idiopathic arthritis has been conducted.

In addition, MIV-711, a cathepsin K inhibitor, significantly alleviated bone and cartilage progression with a reassuring safety profile in two phase 2 trials (NCT02705625, NCT03037489). Denosumab treatment was also demonstrated to inhibit the progression of joint destruction in several trials (Table 4). However, although possessing an anabolic effect by targeting calcitonin receptors on chondrocytes, salmon calcitonin showed no additional benefit in OA treatment in two phase 3 trials (NCT00486434, NCT00704847). TPX-100, a 23-amino acid peptide derived from matrix extracellular phosphoglycoprotein (MEPE) that is highly expressed in osteocytes and downregulated in osteoarthritis, was proven to attenuate progression and induce articular cartilage formation by intra-articular injection in preclinical studies and a few phase 2 trials.^463^ Of note, osteoclast-derived Netrin-1 and osteoclast precursors-derived nerve growth factors (NGFs) can induce nerve innervation in subchondral bone and cause pain during OA development.^464^ Tanezumab, a highly selective immunoglobulin G2 antibody against NGF, has been proven effective and well-tolerated in several phase trials (Table 4). These results suggest the therapeutic potential of targeting subchondral bone remodeling in arthritis.

## Conclusion and perspectives

As a significant activity maintaining skeletal mechanical competence and coordinating the removal of old bone and formation of new bone, bone remodeling is a complex and delicate process mediated by all bone cells, while disruption of a certain link can break the balance and result in disease genesis. Although a few agents have been approved to regulate bone remodeling, here we show that their targets can be further improved to decrease skeletal and extra-skeleton side effects. Meanwhile, with the deepening knowledge of cellular activities during bone remodeling, more therapeutic targets of membrane expression, cellular crosstalk, and gene expression have been exploited with promising therapeutic effects in preclinical studies, which may provide more options to develop bone remodeling agents. In current clinical practice, combination therapies of approved drugs are under investigation for a more extensive and rapid increase in bone mass in severe cases,^465^ which may also be realized by developing novel drug candidates with dual-regulating functions, such as semaphorins and miR-214-3p. In addition, a sequential therapy that starts with bone formation agents, followed by anti-resorption treatment, is also currently emphasized to realize longer-acting bone mass and density maintenance while decreasing the side effects of single anabolic medications or anti-resorption agents.^466^ It may also be achieved by developing novel drug targets, such as LGR4, miR-182, and circBBS9, which have less inhibition of physiological osteoclastogenesis and osteoclast activities that are critical for healthy bone remodeling. Nevertheless, the current comprehension of bone biology and its crosstalk with other systems remains insufficient. The potential influence on the whole bone remodeling process and other systems rather than a particular bone microenvironment or single intercellular interactions are the pendent parts that require further investigations for developing more precise targets.

Since Paul Ehrlich proposed the magic bullet concept, it has been theorized that creating a chemical substance that can specifically attack bad cells without harming the good ones can be possible. For cancers, chemotherapy has raised tremendous hope, but there are still nonnegligible side effects due to the difficulties of making the drugs specific enough only to influence the targeted organ. In skeletal disease therapy, the lack of bone-specific affinity also limits the use and development of many drugs. Such a dilemma triggered the emergence of vector-based drug delivery. In preclinical studies, increasing bone-targeted delivery ligands have been exploited with a pronounced enhancement of bone tissue selection, concentration, and retention time for drugs, which can be promising to solve off-target concerns, especially for small molecule drugs and the emerging oligonucleotide-based gene therapy. Among them, acidic oligopeptides have been approved by the FDA for the preparation of asfotase alfa, an inspiring breakthrough drug in hypophosphatasia treatment, which is a successful clinical translation of bone-targeted delivery ligands. With the promotion of precision medicine, more bone-targeted ligands may emerge in the clinical treatment of bone diseases.

In all, we summarized recent advances in bone remodeling biology, bone-targeted drug delivery, preclinical exploitation, and clinical application of therapeutic agents targeting bone remodeling. We hope this work can help understand and develop novel targeted therapeutic strategies for bone remodeling.
